# varAmpliCNV: analyzing variance of amplicons to detect CNVs in targeted NGS data

**DOI:** 10.1093/bioinformatics/btac756

**Published:** 2022-11-28

**Authors:** Ajay Anand Kumar, Bart Loeys, Gerarda Van De Beek, Nils Peeters, Wim Wuyts, Lut Van Laer, Geert Vandeweyer, Maaike Alaerts

**Affiliations:** Center of Medical Genetics, University of Antwerp/Antwerp University Hospital, Antwerp (Edegem) 2650, Belgium; Biomedical Informatics, Antwerp University Hospital, Antwerp (Wilrijk) 2610, Belgium; Open Targets, Wellcome Genome Campus, Hinxton, Cambridgeshire CB10 1SD, United Kingdom; European Molecular Biology Laboratory, European Bioinformatics Institute (EMBL-EBI), Wellcome Genome Campus, Hinxton, Cambridgeshire CB10 1SD, United Kingdom; Center of Medical Genetics, University of Antwerp/Antwerp University Hospital, Antwerp (Edegem) 2650, Belgium; Center of Medical Genetics, University of Antwerp/Antwerp University Hospital, Antwerp (Edegem) 2650, Belgium; Center of Medical Genetics, University of Antwerp/Antwerp University Hospital, Antwerp (Edegem) 2650, Belgium; Center of Medical Genetics, University of Antwerp/Antwerp University Hospital, Antwerp (Edegem) 2650, Belgium; Center of Medical Genetics, University of Antwerp/Antwerp University Hospital, Antwerp (Edegem) 2650, Belgium; Center of Medical Genetics, University of Antwerp/Antwerp University Hospital, Antwerp (Edegem) 2650, Belgium; Biomedical Informatics, Antwerp University Hospital, Antwerp (Wilrijk) 2610, Belgium; Center of Medical Genetics, University of Antwerp/Antwerp University Hospital, Antwerp (Edegem) 2650, Belgium

## Abstract

**Motivation:**

Computational identification of copy number variants (CNVs) in sequencing data is a challenging task. Existing CNV-detection methods account for various sources of variation and perform different normalization strategies. However, their applicability and predictions are restricted to specific enrichment protocols. Here, we introduce a novel tool named varAmpliCNV, specifically designed for CNV-detection in amplicon-based targeted resequencing data (Haloplex™ enrichment protocol) in the absence of matched controls. VarAmpliCNV utilizes principal component analysis (PCA) and/or metric dimensional scaling (MDS) to control variances of amplicon associated read counts enabling effective detection of CNV signals.

**Results:**

Performance of VarAmpliCNV was compared against three existing methods (ConVaDING, ONCOCNV and DECoN) on data of 167 samples run with an aortic aneurysm gene panel (*n* = 30), including 9 positive control samples. Additionally, we validated the performance on a large deafness gene panel (*n* = 145) run on 138 samples, containing 4 positive controls. VarAmpliCNV achieved higher sensitivity (100%) and specificity (99.78%) in comparison to competing methods. In addition, unsupervised clustering of CNV segments and visualization plots of amplicons spanning these regions are included as a downstream strategy to filter out false positives.

**Availability and implementation:**

The tool is freely available through galaxy toolshed and at: https://hub.docker.com/r/cmgantwerpen/varamplicnv. Supplementary Data File S1: https://tinyurl.com/2yzswyhh; Supplementary Data File S2: https://tinyurl.com/ycyf2fb4.

**Supplementary information:**

Supplementary data are available at *Bioinformatics* online.

## 1 Introduction

Copy number variants (CNVs) are a class of structural variants involving deletion or duplication of specific DNA segments, ranging from 50 to several thousand basepairs (bp), potentially including several genes (MacDonald *et al.*, 2014), and are known to be associated with various diseases including congenital heart disease (CHD) (Costain *et al.*, 2016), Parkinson (Pankratz *et al.*, 2011), diabetes mellitus (Jeon *et al.*, 2010) and autism (Pinto *et al.*, 2010; Shaikh *et al.*, 2009).

Array-based comparative genomic hybridization, fluorescence *in situ* hybridization and single-nucleotide polymorphism (SNP) arrays have traditionally been used to detect somatic (Walter *et al.*, 2009) and germline CNVs (Shaikh *et al.*, 2009). Over the last decade technologies such as multiplex ligation-dependent probe amplification (MLPA) has gained popularity in genetic diagnostics for detection and as a validation tool of CNVs (Stuppia *et al.*, 2012). Most of these tools suffer from poor sensitivity in detecting shorter CNVs such as single exon deletions/duplication events, have high-throughput limitations (except for MLPA) and only a limited number of targets can be analyzed. The advent of next-generation sequencing (NGS) approaches has enabled CNV detection with far greater resolution in comparison to the traditional methodologies. When using targeted resequencing (TR) or whole exome sequencing (WES), the choice of capture protocol for the customized gene panel or exome is important for effective detection of CNVs. There are two main categories of enrichment protocols to capture a given region of interest (ROI): (1) amplicon-based technologies (e.g. Haloplex™ and AmpliSeq™) use oligonucleotides as PCR primers to amplify the targets. Specifically, in the Haloplex™ method, the genomic DNA is fragmented with restriction enzymes and subsequently oligonucleotides complementary to the 5′ and 3′ ends of each fragment are used as PCR primers and (2) hybridization capture-based technologies (e.g. SeqCap™ and SureSelect™) use sonication-based fragmentation to shear the genomic DNA and generate random size DNA fragments. Next, specific oligonucleotide probes are hybridized and used to capture the targeted ROIs (Samorodnitsky *et al.*, 2015).

Computational detection of CNVs from NGS data is a challenging task. In recent years, many new methods have been developed and applied on a varied range of datasets (Bellos *et al.*, 2014; Boeva *et al.*, 2014; Derouault *et al.*, 2020; Fowler *et al.*, 2016; Johansson *et al.*, 2016; Li *et al.*, 2012; Povysil *et al.*, 2017; Talevich *et al.*, 2016; Tan *et al.*, 2014; Zhao *et al.*, 2013). These methods are based on five different strategies: (i) read depth (RD), (ii) paired-end mapping, (iii) split read, (iv) *de novo* assembly and (v) combinations of any of the above approaches (Zhao *et al.*, 2013). Among these strategies only RD-based approaches can be successfully applied to WES or TR while all are applicable to whole-genome sequencing (WGS) data (Tan *et al.*, 2014). The main reason being that the ROI in WES and TR is only a fraction of that in WGS. Additionally, RD-based approaches incorporate the counting of number of reads aligned to a given ROI that has been empirically determined to be proportional to the number of copies of genomic segments, which helps in directly quantifying the CNVs with respect to RD.

Computational detection of CNVs through RD requires normalization of the input data such that variability of RD is minimized, followed by detection of CNVs by comparing with control samples processed in a similar way. The normalization procedure includes accounting for biases associated to the enrichment protocol, non-uniform depth of coverage across the ROI and sequence properties such as local GC content and presence of repetitive elements. Most of the existing methods take these biases into account in their analysis pipelines. For example, ONCOCNV (Boeva *et al.*, 2014) and CovCopCan (Derouault *et al.*, 2020) were developed and tested on samples subjected to amplicon-based enrichment (AmpliSeq™). For ONCOCNV, the read counting is done at the amplicon level in the sense that each read is assigned to the amplicon it overlaps most with. In case of overlapping amplicons, they are merged if overlapping more than 75%. It then subsequently incorporates multi-factor tumor sample normalization for potential enrichment biases and noises, and then performs principal component analysis (PCA) on a set of normal controls to extract the baseline coverage. Subsequently, segmenting the logarithmic ratio between the tumor and normal samples gives the putative CNVs. Similarly, CoNVaDING (Johansson *et al.*, 2016) was developed specifically for hybridization-based (SureSelect™) TR data to detect single exon copy number events. The normalization procedure involves two stages where in a first step the data are normalized using all targets within the sample and a second step uses all targets within the gene. Additionally, it incorporates stringent quality control metrics to select the most informative control samples such that distribution patterns of RD are highly similar between query and control samples used to compute the coverage ratio. Another tool called DECoN (Fowler *et al.*, 2016) adapts the ExomeDepth (Plagnol *et al.*, 2012) package, which internally fits a beta-binomial model to describe the read distribution of the samples to detect exon copy number variations. Originally, it was designed and validated on hybridization-based enrichment protocols.

Many of these tools give robust performance on their own benchmark dataset, with high sensitivity and specificity (Moreno-Cabrera *et al.*, 2020). However, their specificity declines dramatically when they are applied on gene panels enriched with different technologies, leading to a large number of false positive (FP) CNV calls which can be attributed to the protocol-specific internal design pattern of enrichment probes (Samorodnitsky *et al.*, 2015; Tan *et al.*, 2014). To our knowledge however, currently no method exists which can be applied directly on Haloplex™-based amplicon sequencing data, which by design result in multiple overlapping amplicons of highly variable length. The resulting RD profiles show a wide range in coverage, and complex overlapping patterns make ONCOCNV unsuitable for HaloPlex™ data.

Hence, we introduce a novel tool called varAmpliCNV, specifically designed to detect germline CNVs in Haloplex™ enriched panel data by analyzing the variance of depth of coverage of individual amplicons. The internal design principle of varAmpliCNV harnesses the amplicon design information and accounts for the overall RD variability using PCA or metric multi-dimensional scaling (MDS). VarAmpliCNV workflow is scalable in design to process large sets of samples. Finally, varAmpliCNV provides unsupervised clustering and visualization of each predicted copy number segment, by plotting RD profiles of individual amplicons in the context of the amplicon design pattern. This clustering and visualization serve as a *post hoc* filter for FP CNV calls.

## 2 Materials and methods

### 2.1 Validation sets

#### 2.1.1 Thoracic aortic aneurysm and dissection panel

TR of a panel of 30 thoracic aortic aneurysm and dissection (TAAD) genes (see Supplementary File S1: sheet 8) using Haloplex™ enrichment was performed on 167 samples divided into five batches according to different sequencing experiments. Nine samples contained a CNV previously validated by MLPA or multiplex amplicon quantification (MAQ) assays; including two full gene duplications and a single exon duplication in the *MYH11* gene, four multiple exon deletions/duplications and a single full gene duplication of *FBN1*, and one two-exon duplication in *TGFBR2* (see Supplementary File S1: sheet 9). All samples were analyzed in a blinded fashion (for the known CNVs) using varAmpliCNV and three other competing methods. Each batch was analyzed independently to avoid any batch-specific biases in the analysis.

#### 2.1.2 Deafness panel

TR of a panel constituting of 145 genes involved in deafness (see Supplementary File S2: sheet 5) after Haloplex™ enrichment was performed on 138 samples divided into four batches. Four samples across these batches contained a SNParray or MAQ assay validated full gene deletion of *OTOA*, *POU3F4*, *EYA4* and *EYA1*, respectively. The main objective of this additional dataset was to validate the thresholds for deletion and amplification derived from the TAAD panel.

### 2.2 VarAmpliCNV workflow

**Fig. 1.**
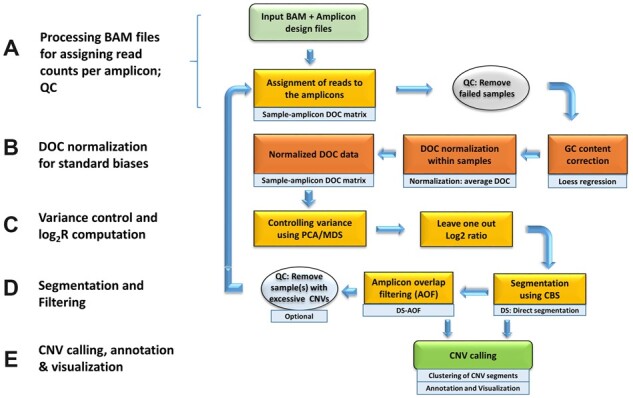
Workflow of varAmpliCNV. (**A**) BAM files are processed to obtain read counts per amplicon. Subsequently, QC metrics are applied to remove bad quality samples from the analysis. (**B**) Read counts are normalized for average depth of coverage (DOC) and GC corrected. Normalization is performed separately for autosomal and sex chromosome targets. (**C**) Remaining variance is reduced using PCA/MDS, followed by a leave-one-out approach to compute Log_2_R ratios per amplicon. (**D**) Detection of CNVs using CBS, optionally followed by AOF. (**E**) Clustering, annotation and visualization of CNV segments

VarAmpliCNV analysis consists of five stages (Fig. 1): (i) Processing the input binary alignment map (BAM). files using the amplicon design to obtain read counts per amplicon. Samples with low average coverage (<100 reads/amplicon) are filtered out, (ii) normalizing the read counts for enrichment biases, (iii) controlling variance using PCA or MDS and subsequent Log_2_R computation, (iv) segmentation of the Log_2_R profile and filtering CNV segments on QC metrics and (v) clustering, annotation and visualization of predicted CNV segments.

#### 2.2.1 Input files

VarAmpliCNV uses BAM files and a target exonic regions (TERs) file (amplicon design file in BED format). The BAM files were obtained from raw *fastq* reads using an in-house pipeline customized for Haloplex™ enrichment data (hg19/GRCh37; Supplementary Fig. S1 in Proost *et al.*, 2015). The TER contains the individual amplicon coordinates spanning all given exons (±51 bp exonic region) of TAAD or deafness panel genes. This corresponds to 4701 and 37 383 amplicons in the TAAD and deafness panels, respectively. A visualization of the structure of the design file illustrates the overlapping amplicon structure and non-uniform distribution of amplicon lengths in Supplementary Figure S1.1A.

#### 2.2.2 Read counting

The aligned reads are uniquely assigned to amplicons by exactly matching the respective start and end coordinates (using python-PySAM package). Our approach removes the impact of mutual dependency due to overlap between the amplicons (see the information flow in the dependency model in Supplementary Fig. S1.1B). Consequently, unstable amplicons will not impact the signal of overlapping amplicons. Furthermore, it reduces the noise arising from unassigned reads, which are typically artifacts from aspecific amplifications. The read count is stored as a read count matrix and by convention we represent the amplicons as rows and samples as columns. Here, each amplicon is considered as an independent data point, defined by the start coordinate. Since for both panels the TR was performed at an average coverage of 4000×, we expect for each sample to have an average coverage of at least 100 reads per amplicon. We removed all samples from the analysis that did not meet this criterion. Similarly, amplicons without assigned reads across all samples were pruned out from the read count matrix.

#### 2.2.3 Read count normalization

The read count data are corrected for biases associated to local GC-amplicon content using Loess-based linear regression (Benjamini and Speed, 2012; Boeva *et al.*, 2014). The GC-corrected matrix was separated for autosomal and sex chromosomes. Normalization for average coverage of the sample is accomplished by dividing the read counts of each amplicon by the average coverage within a given sample.

#### 2.2.4 Controlling variance

The most fundamental problem of all existing methods is to control the variance of coverage in the targeted regions. We address this issue by formulating an objective function which can transform the normalized read count matrix in such a way that variance is minimized. This is accomplished primarily by PCA. Using PCA, we estimate the orthogonal principal components (PCs) and arrange them according to the proportion of variance they explain. We chose to remove those PCs that account for 80% of the variance (see results for evaluation of this choice). This reverse denoising step is computed using Equation (9) of Supplementary Section S3.1. Since PCA can become computationally intensive with number of amplicons, we additionally implemented an alternative approach based on MDS, providing identical results with faster execution time. To obtain identical results with MDS and PCA (Cox and Cox, 2000), it is required that the Euclidian distance measure between the data points (amplicon with read counts) should be incorporated. Using the Euclidean distance is apt for our data because it is non-spatial and does not represent any three-dimensional coordinate system (Supplementary Section S3.2).

#### 2.2.5 Computation of ratio score (Log_2_R)

All existing methods require a matched control dataset for comparing the normalized RD of a sample using ratio scores. The logarithm of this ratio score (Log_2_R) gives a distinctive negative value for a deletion and a positive value for an amplification. When a matched control sample is not available, this can be simulated by pooling normal samples. In our case, a leave-one-out approach selects the reference samples. This means that for each amplicon, the denominator of the ratio of the normalized read counts is computed by taking the average normalized read count of the remaining *n*−1 samples (where *n* is the total number of samples passing QC present in a given experiment).

#### 2.2.6 CNV calling using segmentation and filtering

Segmentation is applied directly on Log_2_R values to detect change point events. For varAmpliCNV, circular binary segmentation (CBS) (Olshen *et al.*, 2004) available from the DNACopy package (Seshan and Olshen, 2014) in R is incorporated. The user can select either (i) direct segmentation (DS) using CBS alone or (ii) by applying a post-processing filtering on CBS-segmented CNVs, called amplicon overlap filtering (DS–AOF).

For DS, the CBS algorithm with default settings is used, which eventually results in a list of putative CNV segments. Segments with Log_2_R values below −0.5 are reported as deletions and those with values above 0.5 are reported as duplications. Additionally, we discard segments covered by less than 10 amplicons or having a standard deviation (SD) greater than 1.

For the DS–AOF approach, information related to the overlapping structure of amplicons is used to recalculate average logarithmic ratios of CNVs predicted by DS. The recalculated segment Log_2_R is a weighted average, where the Log_2_R of each amplicon overlapping the segment contributes relative to the number of overlapping positions. The conceptual details of this approach can be found in Supplementary Section S4.

#### 2.2.7 Annotation and validation of CNV segments

The output format is a tab-separated list of predicted CNV segments with sample names, coordinates and summary statistics. Segments are annotated with gene names, exon numbers and number of involved amplicons which are provided by the user. Additionally, each CNV is visualized through plots, presenting the TERs, gene and corresponding aligned amplicon structure (see Supplementary Section S4.2).

In addition, a sample-specific QC metric was formulated related to these predicted CNV segments, discarding samples containing a significantly higher number of CNVs than generally expected. Significance was evaluated using Student’s *t* test, comparing the CNV count of each sample against the remainder of the batch. If any samples are discarded due to this, QC, PCA/MDS, Log_2_R calculation and segmentation steps are repeated to exclude the impact of low-quality samples on the reference.

In the TAAD panel analysis, all CNV segments predicted by varAmpliCNV and passing filtering on SD ≤1 and amplicon number count ≥10 were experimentally evaluated using MLPA/MAQ assays. Additionally, CNVs predicted by at least two competing methods and missing/overlapping with varAmpliCNV predicted CNV segments were also validated using MLPA/MAQ assays (see Supplementary File S1: sheet 6). Based on the validation results, we evaluated the sensitivity and specificity performance of the tool.

#### 2.2.8 Clustering analysis for performance evaluation

The initial analysis was done on TAAD panel data in a blindfolded manner, where only post-CNV predictions of the true positives (TPs) were revealed. For sensitivity and specificity analysis, default parameter settings of the competing methods were used. For varAmpliCNV, which is based on a two-step prediction strategy (PCA/MDS and DS/DS-AOF), we applied default settings: (i) removal of approximately 80% of proportion variance in PCA/MDS step, (ii) filtering of CNV segments with SD ≤ 1 and amplicon number count ≥10 and finally, (iii) filtering of CNV segments using a segmentation threshold (ST), a primary cut-off for the average normalized Log_2_R ratio of a CBS-generated segment ≥0.50 (for duplication) or ≤−0.50 (for deletion).

Next, a data-driven unsupervised clustering approach is applied on the predicted CNV segments to detect clusters of TPs and FPs. Specifically, we incorporate an R package ‘Ckmeans.1d.dp’ (Wang and Song, 2011) which utilizes an optimal *k*-means 1D clustering using dynamic programming to cluster CNV segments. The predicted CNV segments were clustered on Log_2_R and average-Log_2_R score for DS and DS–AOF approach, respectively. The choice of number of clusters ‘*k*’ was determined empirically (data driven) using maximum Bayesian information criterion (BIC) score as suggested in the package manual.

## 3 Results

### 3.1 TAAD panel data analysis

#### 3.1.1 Sample selection

In total, 167 samples, corresponding to five experimental batches, are processed to obtain amplicon-based read counts. Samples having average read counts less than 100 were excluded from the analysis. Six, one, zero, one and two samples were excluded from the respective batches, resulting in a total count of 157 samples.

#### 3.1.2 Effect of GC content

In our analysis pipeline, no significant correlation between GC content and RD is observed. Supplementary Figures S2.1 and S2.2 demonstrate that for all batches the average correlation is almost zero for autosomal target regions and approximately 0.20 for sex chromosomal targets. However, we still correct for this effect using Loess-based measure, as correlation might be higher in other target regions.

#### 3.1.3 PCA/MDS-based normalization and usage of AOF

The varAmpliCNV pipeline controls for the variance in the depth of coverage of TR panel data by performing PCA/MDS, with removal of an optimal number of PCs. The number of removed PCs is batch specific, since each of them is analyzed independently. Applying default setting of approximately 80% variance removal corresponds to removal of 0 to 4PCs leading to removal of a proportion of variance ranging from 56% to 96% across five batches (see Supplementary File S1: sheet 7). All TPs are correctly retained in the data when we remove approximately 80% of variance. Additionally, in the analysis pipeline, we account for removal of samples containing an excessive number of CNVs. In the first (s25) and second batch (s14 and s25) there were three samples, behaving aberrantly and containing an unexpectedly high number of CNVs. After removal of these samples, the analysis was repeated from the beginning (PCA/MDS, Log_2_R calculation and segmentation).

The proportion of variance explained by the removed number of PCs for each batch was 82.22%, 85.19%, 77.21%, 0% (no PCs were removed in this case) and 78.17% for the autosomal targets. Details of all CNV segments corresponding to varying numbers of removed PCs can be found in Supplementary File S1: sheets 1–5 for all the five batches. This resulted in a total of 15 CNV segments (T1–T15) (see Supplementary File S1: sheet 6).

**Fig. 2.**
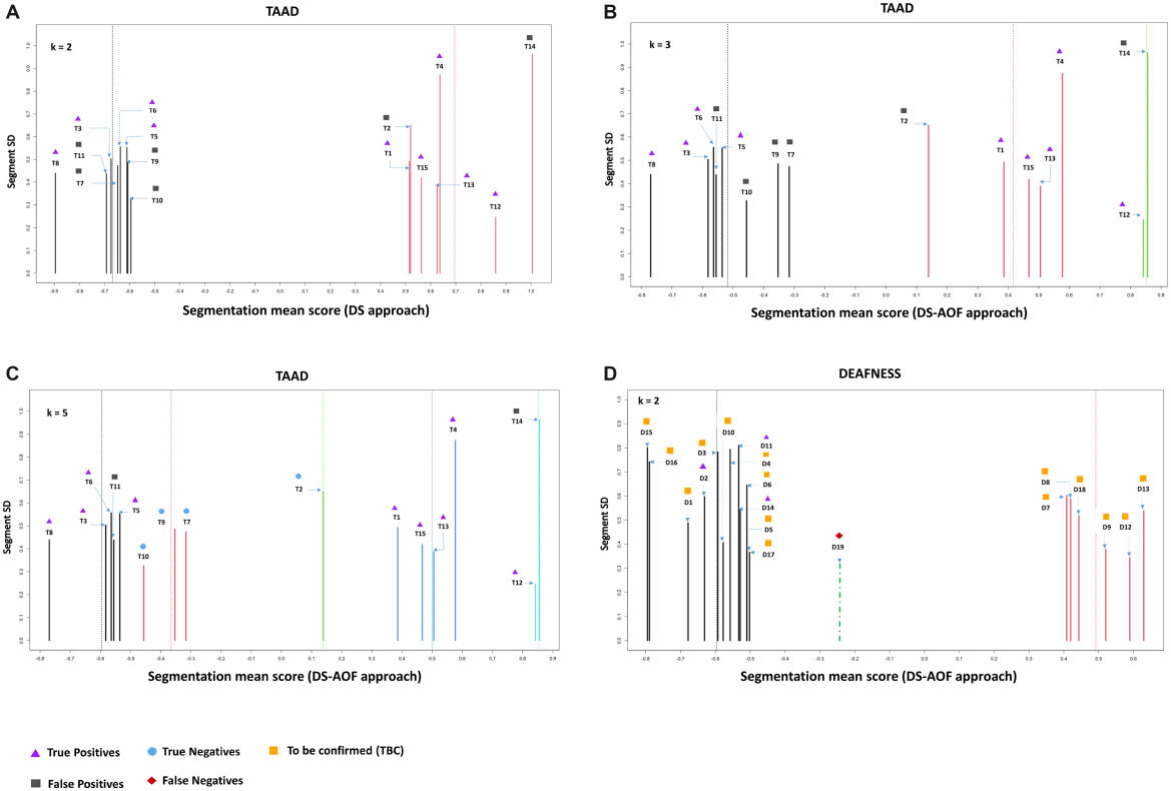
Evaluation of CNVs using DS, and AOF (DS–AOF) with *k*-means clustering: CNV segment data points clustered using 1D *k*-means algorithm. The horizontal axis represents the CNV segments mean Log_2_R score and vertical axis represent the CNV segment SD. TAAD panel: (**A**) DS approach on 15 CNV segments yielded two clusters (max BIC score) having cluster means (−0.67, 0.69) resulting four FPs (T7, T9, T10 and T11 deletions) and two FPs (T2 and T14 amplifications) clustered along with TPs. (**B**) DS–AOF approach on CNV segments yielded three clusters (max BIC score) resulting four FP deletions and two FP amplifications clustered along with TPs. (**C**) DS–AOF approach with selected choice of *k* = 5 clusters resulted in optimal clustering of CNVs where previous FPs (T2, T7, T9 and T10) can now be classified separately. Only two FPs get clustered along with the TPs. Deafness panel: (**D**) DS–AOF approach on 18 CNV segments resulted in *k* = 2 (max BIC score) clusters where three TPs got clustered together along with other candidate CNVs (TBC) which need to be validated using orthogonal methods (MLPA/MAQ). These TBC CNVs can be further filtered by annotation and visualization plots (see Section 3.4, Fig. 3 and Supplementary Section S4.2). CNV segment D19 is a FN and missed in the candidate CNV list because of ST cut-off ]−0.5, +0.5[

Next, the *k*-means 1D cluster analysis of predicted CNV segments resulted in two clusters for the DS approach as shown in Figure 2A. The optimal choice of *k* = 2 clusters was determined empirically using the BIC score as shown in Supplementary Figure S5.1. The first cluster corresponds to all CNV deletions, encoded by black vertical bars encompassing four TPs (T8, T3, T6 and T5) and four FPs (T11, T7, T9 and T10). The second cluster corresponds to all CNV duplications, encoded by red vertical bars encompassing five TPs (T1, T15, T13, T14 and T12) and two FPs (T2 and T14). The cluster means are −0.67 and 0.69 (indicated with dashed vertical line) corresponding to clusters of the deletions and duplications, respectively.

Similarly, with DS–AOF approach-based predicted CNV segments, the cluster analysis resulted in optimal *k* = 3 clusters (max BIC score; Supplementary Fig. S5.2) encoded by black (deletions), red and green (duplications) with respective cluster means −0.52, +0.42 and +0.85 as shown in Figure 2B. The black deletion cluster encompasses four TPs (T8, T3, T6 and T5) and four FPs (T11, T7, T9 and T10). For CNV duplications, the red cluster comprises four TPs (T1, T15, T13 and T4) and one FP (T2) and the green cluster contains one TP (T12) and one FP (T14).

Additionally, when the optimal number of clusters is chosen as *k* = 5, this results in five distinct clusters encoded by black, red, green, blue and aquamarine having cluster means −0.60, −0.37, +0.14, +0.50 and +0.85 respectively, as shown in Figure 2C. It can be observed that there are two distinct clusters for deletions. The black cluster encompasses four TPs (T8, T3, T6 and T5) and one FP (T11). The red cluster comprises three true negatives (TNs) (T10, T9 and T7). For the duplications, there are three clusters. The first green one is a singleton containing one TN (T2). The blue cluster comprises four TPs only and finally the aquamarine-colored cluster comprises one TP (T12) and one FP (T14).

It can be deduced from Figure 2A–C that application of the DS–AOF approach results in more distinct clusters in comparison to the DS approach thereby optimizing TP, FP and TN detection.

### 3.2 Performance comparison with ONCOCNV, CoNVaDING and DECoN

The predictive performance of varAmpliCNV, ONCOCNV(v6.6), CoNVaDING (v1.2.1) and DECoN (v1.0.1) was evaluated on nine known CNVs in the TAAD gene panel (see Supplementary File S1: sheet 9). Apart from the 15 CNVs predicted by varAmpliCNV, all other methods predicted many CNVs (Table 1), making individual validation practically infeasible. Hence, for the competing methods, only a subset of 21 CNVs congruent between at least two methods was validated (see Supplementary File: sheet 6). So overall, 36 CNVs were validated using MLPA/MAQ assays of which 9 were confirmed as TPs. We determined the sensitivity and specificity of these tools by calculating the number of TPs, FPs, false negatives (FNs) and TNs. When the known validated CNVs were correctly predicted in the blindfolded analysis, these are classified as TPs. Predicted CNVs that could not be confirmed by orthogonal methods (MLPA/MAQ assays) are classified as FPs. Subsequently, known CNVs that were not predicted are classified as FNs. Finally, all given autosomal and sex chromosomal targets (each exon is defined as a target) for which no CNV calls were made by the method were marked as TNs. Sensitivity was calculated as #TP/(#TP + #FN) and specificity as #TN/(#TN + #FP). The analysis results of varAmpliCNV and competing methods are presented in Table 1. The list of all the CNVs analyzed in this performance analysis is presented in Supplementary File S1: sheet 6.

**Table 1.** Performance comparison of varAmpliCNV with ONCOCNV, CoNVaDING and DECoN on the TAAD panel: The counts for TPs and FPs for varAmpliCNV (using DS and DS–AOF) were obtained using the ST threshold of ]−0.50, +0.50[ in combination with clustering and visualization

Performance on the TAAD panel	Total CNV calls	CNVs for validation	TPs	FPs	FNs	TNs	**Sensitivity** **[CI] (%)**	**Specificity** **[CI] (%)**
varAmpliCNV (DS) clustering (BIC *k* = 2)	15	15	9	6	0	458	100 [66.37–100]	98.70 [98.11–99.87]
varAmpliCNV (DS) clustering (BIC *k* = 2) and visualization	15	15	9	3	0	458	100 [66.37–100]	99.31 [98.11–99.87]
varAmpliCNV (DS–AOF) clustering (BIC *k* = 3)	15	15	9	6	0	458	100 [66.37–100]	98.70 [97.21–99.52]
varAmpliCNV (DS–AOF) clustering (BIC *k* = 3) & visualization	15	15	9	3	0	458	100 [66.37–100]	99.31 [98.11–99.87]
varAmpliCNV (DS–AOF) clustering (*k* = 5) and visualization	15	15	9	1	0	458	100 [66.37–100]	99.78 [98.79–99.99]
ONCOCNV	41	15	6	6	3	255	66.67 [29.93 –92.51]	97.70 [95.06–99.15]
CoNVaDING	445	15	3	6	6	175	33.33 [7.49–70.07]	96.68 [92.92–98.77]
DECoN	291	18	9	9	0	403	100 [66.37–100]	97.81 [95.89–99.00]

*Notes*: For ONCOCNV, CoNVaDING and DECoN, the analysis was done with their respective default settings. For the competing methods, sensitivity and specificity along with respective confidence intervals (CIs) were calculated for validated CNVs only.

### 3.3 Application of DS–AOF on the deafness panel data

Applying the default settings of varAmpliCNV and using the DS–AOF approach, we predicted CNV segments for another gene panel related to deafness, run on 138 samples grouped into four batches. It resulted in 19 CNV segments as described in Supplementary File S2: sheet 5. Three out of four previously validated CNVs (using SNP-array) were among this list of 19 CNVs and thus were successfully predicted. One deletion present in Batch 4 was not detected with the current threshold settings. The remaining predicted 15/19 CNV segments were re-inspected using the commercial software package SeqPilot (JSI medical systems) but could not be confirmed in the visualized read data and are more likely to be FPs. However, in order to correctly classify them as FPs, orthogonal methods-based validation (MLPA/MAQ, similar to TAAD panel) is required. Hence, at this moment, we assign them for further testing to be confirmed (TBC). The overall results are summarized in Figure 2D and Supplementary File S2: sheet 5.

### 3.4 Filtering via annotation and visualization

**Fig. 3.**
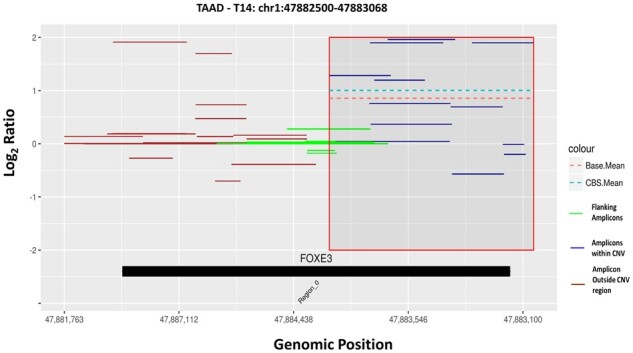
Evaluating CNV segments by visualization plots. (**A**) For the TAAD panel, visualization plot of data point T14 (FP) in batch4 from Figure 2A–C, confirmed as FP by orthogonal methods. (**B**) For the deafness panel, eight data points (as shown in Fig. 2D) were filtered out thereby trimming the list of TBC by 53.33%

Using varAmpliCNV results in candidate CNV segments TBC by orthogonal methods. This candidate list can be trimmed by evaluating the representation and density of the amplicons in and around the predicted CNV region and its overlap with the TERs of genes in the panel. This can be best visualized through plots where all this information is geometrically embedded, thereby easing out the evaluation. For example, in the TAAD panel data point T14 is a confirmed FP clustered together with TPs in clusters with color red (Fig. 2A), green (Fig. 2B) and aquamarine (Fig. 2C). However, after annotating the predicted CNV segment to the TER: Region_0 (mapped Exon 1) of *FOXE3* along with the list of all amplicons aligned to this region, it is observed to be a potential partial exonic duplication as shown in Figure 3 (also see Supplementary Section S4.2.1). Similarly, CNV segments T2, T7 and T9 are partial exonic CNVs (see Supplementary Fig. S4.4, S4.9 and S4.11). For Haloplex™ data, we limit our resolution to predict single exon CNVs and any such partial deletion/duplication of a single exon can be classified as FP. Based on this idea, we were able to remove eight CNV segments that were partially deleted/duplicated from the candidate list of CNVs TBC in the deafness panel (see Supplementary Section S4.1.3, pp. 32–33). As such the list of CNVs TBC was reduced to 7 out of 15 CNV segments predicted by the DS–AOF approach, removing 8 data points (D1, D3, D6, D8, D9, D13, D17 and D18) from Figure 2D (approximately 53% trimming).

## 4 Discussion

CNV detection in TR data is a challenging task and it is limited due to variability in the high RD coverage, biases associated with specific enrichment protocols and lack of matched controls especially in clinical diagnostic settings. We have developed varAmpliCNV, a novel approach toward predicting CNV segments from TR sequencing data, designed specifically to handle amplicon-based sequencing NGS data enriched by Haloplex™ technologies. VarAmpliCNV incorporates a unique two rounds strategy to model the flow of information from RD count to the genomic position that is mediated by the overlapping amplicons. In the first round, reads are uniquely assigned to the amplicons. Then it implements PCA/MDS-based methodology as normalization to control the variance of RD associated with the amplicons which upon segmentation eventually leads to putative CNV segments. In the second round, it utilizes the dependencies between the amplicons to filter out the potential FP segments thereby detecting CNVs with higher sensitivity and specificity in comparison to competing methods.

For our validation dataset, which consists of high-coverage NGS data from targeted TAAD gene panel, the initial blindfolded analysis using varAmpliCNV retrieved all the known TP CNVs with high sensitivity (100%) and specificity (99.56%) in comparison to the three competing methods. ONCOCNV, which was also designed for handling amplicon sequencing data on targeted cancer gene panel, achieved much lower sensitivity (66.67%) and slightly lower specificity (97.70%) scores. Performance of DECoN in detecting all TPs was on par with that of varAmpliCNV thereby achieving 100% sensitivity but comparatively it detected slightly more FPs leading to a lower specificity (97.81%). Finally, CoNVaDING, which was primarily designed to detect single exon CNV events in amplicon-specific TR data, achieved the lowest sensitivity (33.33%) and specificity (96.68%) among all the competing methods. In addition, the competing methods predicted many more CNVs (41–445) compared with VarAmpliCNV (15). This leaves many more CNVs to be confirmed and if these turn out to be FPs, specificity would be much lower (87.93% for ONCOCNV, 58.83% for DECoN and 28.36% for CoNVaDING). This analysis clearly indicates the poor applicability of the existing methods for reliably predicting CNVs from Haloplex™-based amplicon sequencing data. It should be noted that for the competing methods, default settings were used and we did not evaluate whether they could better adapt to the dataset by modifying their thresholds.

From Table 1, it can be deduced that the DS and DS–AOF approach have equivalent performance for sensitivity and specificity using data-driven optimal number of clusters (*k* = 2 and 3, respectively). However, the impact of the DS–AOF approach (Fig. 2B) can meaningfully be observed where the FPs (T2, T7, T9 and T10) start moving away from clusters of TPs. Eventually, with increase in number of clusters (*k* = 5, Fig. 2C) all the FPs can distinguishably be classified in a separate cluster. This signifies the utility of the AOF approach in optimal detection of germline CNVs.

Additionally, denoising the normalized RC using PCA/MDS has distinct utility in reducing the signal-to-noise ratio thereby enhancing true CNV signal (Supplementary Section S3.3, Fig. S3.1) as shown for TP controls pertaining to Batch3 of the TAAD panel.

Current state-of-the-art methods are robust in handling standard sequence-specific biases, but show limitations in handling variability associated with high depth of coverage. For example, ONCOCNV incorporates PCA only to capture the baseline coverage using a set of PCs (by default first three PCs) from the matched control set to compute ratio scores. In contrast, varAmpliCNV has been designed to predict CNVs in absence of a matched control set. Hence, the principle of PCA is applied directly on all the samples in a single batch to minimize or control the variability of read counts. Next, enrichment designs are typically handled specifically for the targeted protocol. In ONCOCNV, offering the closest match to the varAmpliCNV methodology, the read counts per amplicon are generated by assigning reads to the amplicon to which it maximally overlaps. In case of more than 75% overlap between amplicons (tiled design pattern amplicons in AmpliSeq™), they are merged into a single amplicon. In varAmpliCNV, the read counts are generated by uniquely assigning the reads directly to each amplicon by exact matching of the start and end coordinates. This results in the removal of low quality, unmapped or partially overlapping reads, which increases the signal-to-noise ratio. Moreover, an MDS-based approach has been incorporated in the workflow, giving identical results as with PCA, but eventually increasing the prediction speed several folds so varAmpliCNV is suitable to handle large sets of amplicons.

Similarly, varAmpliCNV outperformed DECoN, which achieved the same sensitivity, by detecting a smaller number of FPs achieving higher specificity. The essential feature of DECoN is that it incorporates functionalities of ExomeDepth package in R to predict CNVs. Internally, ExomeDepth models the RD count ratio using a beta binomial distribution that accounts for its over-dispersion. Comparatively, varAmpliCNV uses a non-parametric approach by using PCA/MDS method without fitting any predefined standard distribution to control the variances in the RD count across the samples. Although both of these methods have equal sensitivity in predicting CNVs, varAmpliCNV performs better in specificity primarily because it utilizes the overlapping structure of amplicons which eventually helps in better clustering of CNVs thereby pruning out the majority of FPs.

Finally, varAmpliCNV outperforms CoNVaDING both in terms of sensitivity and specificity. The internal design principle of CoNVaDING is based on a comparison of the distribution score of target read count with a set of matched controls. It also ignores the variability associated with the underlying enrichment design protocol. This is evident from the fact that read counts are generated at the exon level and leads to prediction of CNVs per targets (TERs), resulting in a long list of CNVs that could be potential FPs.

Although the sensitivities of recently developed methods are increasing, controlling the specificity (the number of FPs) attributes as the limiting factor regarding the applicability of these tools for reliable and usable prediction. The competing methods do not incorporate post-processing steps for FP pruning. VarAmpliCNV provides a unique novel strategy called AOF in conjugation with unsupervised clustering and visualization plots to deal with filtering of FPs. Principally, the AOF functionality can also be extended toward other amplicon-based sequencing technologies such as AmpliSeq™. Together, the clustering and graphical representation enable non-computational users to explore the predicted CNV segments and then optimally decide on a selection for wet lab validation and subsequent clinical reporting.

In spite of achieving high sensitivity and specificity there are some potential limitations to varAmpliCNV. Foremost, the default ST cut-off boundaries for deletion and duplication ]−0.5, 0.5[ might miss some interesting CNV segments whose score might fall outside this range. Hence, generalizing the applicability of the same cut-off values on other targeted panel data could lead to potential FNs. As a proof of concept, we applied the derived cut-offs on an independent deafness panel. We have shown that three-fourth known CNVs (SNP-array validated) could be identified correctly, but a single full gene deletion was not detected. A possible reason for this missed deletion might be the lower overall average coverage (computed in the normalization step B of the workflow) of the sample containing this CNV in comparison to other samples. This can lead to skewing of the estimation of proportion of variance in PCA/MDS, leading to a loss of true signal during noise removal. In such lower quality scenarios, we recommend inspecting both the raw CNV list and the filtered CNV list (DS/DS–AOF approach).

Secondly, users can remove less than the recommended 80% variance, such that the true signal is retained at the cost of some additional FPs. In addition to the high sensitivity, we obtained only 15/19 CNV segments as the list of candidates to be confirmed with other methods. We further utilized varAmpliCNV’s annotation and visualization plots to trim down this list. This resulted in removal of 8/15 candidate CNVs, leaving only 7 CNV segments to be validated. This number is very low in comparison to the long lists of CNVs reported by the evaluated competing methods analyzing data of the TAAD panel, containing just a fourth of the genes compared with the deafness panel. Finally, for 1D *k*-means clustering of the segmentation score, the choice of optimal number of clusters is crucial and users may seek other methods comparable to BIC score plots to filter the FPs. The combination of DS–AOF with unsupervised clustering and filtering by visualization contributes toward higher specificity of varAmpliCNV thereby making it feasible for wet-lab validation and clinical diagnostic reporting. Incorporation of additional information, such as the allele frequencies of informative heterozygous SNVs present in the predicted CNV segments, can further aid in discriminating TP and FP CNVs.

## 5 Conclusions

The varAmpliCNV workflow provides a modular approach to handle variability and complexities in amplicon sequencing data, in the context of CNV prediction. The information granularity, flowing from sequencing reads to exon-level targets is indirectly mediated by the enriched amplicons. Leveraging the design information enables us to capture underlying dependencies in the data. The presented PCA/MDS-based method captures variability at the individual amplicon level, while the positional dependency between the amplicons and subsequent annotation and visualization plots are used to filter out FPs. VarAmpliCNV is easy to use via command line and Galaxy. The analysis results can be visualized, making interpretation straightforward for both bioinformaticians and lab technicians. Together, varAmpliCNV presents a novel approach in detecting germline CNVs with high sensitivity and specificity on amplicon sequencing data applicable in both research and clinical diagnostic settings.

## Supplementary Material

btac756_Supplementary_Data
