# Supplementary material for: varAmpliCNV: analyzing variance of amplicons to detect CNVs in targeted NGS data
Source: Bioinformatics. 2022 Nov 28;39(1):btac756. doi: 10.1093/bioinformatics/btac756 (PMC9805572; doi:10.1093/bioinformatics/btac756)
Supplement: btac756_Supplementary_Data [file btac756_supplementary_data.pdf]

varAmpliCNV: Analyzing Variance of Amplicons to detect CNVs in targeted NGS data - Supplementary Data

Ajay Anand Kumar, Bart Loeys, Gerarda Van De Beek, Nils Peeters, Wim Wuyts, Lut Van Laer, Geert Vandeweyer*, Maaike Alaerts*

S1 Information flow for the amplicon design patterns

In a Haloplex-based targeted gene panel the region of interest (ROI) or targeted exonic region (TER) is covered by amplicons in a unique overlapping mesh-like structure. This overlapping structure allows for characterization of CNVs with good precision and accuracy. An example representation of this structure is shown in Figure S1.1 (chr15:48702743-48704045) for one of the targeted region of *FBN1* gene. The reads are generated with respect to each of the amplicons with discrete start and end coordinates. Hence, the flow of information can be described from read count to the targeted ROI via the amplicons. This flow can be encoded using the graphical structure as shown in Figure S1.1B. The given graph has directed acyclic structure with nodes and directed edges. In the top there is single node representing the Read Count (RC). The amplicons $A_1, A_2, A_3, ..., A_n$ represent the set of amplicons aligned for given ROI thereby constituting the intermediate layer. Finally, the targeted positions $P_1, P_2, P_3, ..P_m$ or ROIs form the bottom layer of the graph. The dependencies in this graph can be described in two ways:

1. The amplicons are independent of each other with respect to the read counts, so read counts can be assigned uniquely to amplicons based on start and end coordinates. Each amplicon is treated as a unique data point, facilitating the application of PCA/MDS and other subsequent steps for CNV detection in the pipeline.

2. The amplicons are dependent on each other with respect to their position, as they form an overlapping structure as shown in Figure S1.1A. For example, position P1 is encompassed by amplicon A_1, A_2 and A_3. Similarly, position P_2 is encompassed by amplicon A_2, A_3 and A_4. This dependency is incorporated as filtering step in AOF (discussed in section S4) to prune out false positives (FPs).

With this graphical structure it is much easier to implement various steps of varAmpliCNV pipeline. The independency with respect to read counts enables assignment of reads to each amplicons matching their start and end coordinates respectively thereby treating each amplicon as unique data point. This helps in applying PCA/MDS and other subsequent steps for CNV detection in the pipeline (see lower part of graphical structure of Figure S1.1B). The dependency of amplicons with respect to position is incorporated as filtering step (AOF) to prune out false positives (see upper part of the graphical structure Figure S1.1B). Overall, encoding of this dependency structure of the amplicons is core of the varAmpliCNV pipeline.

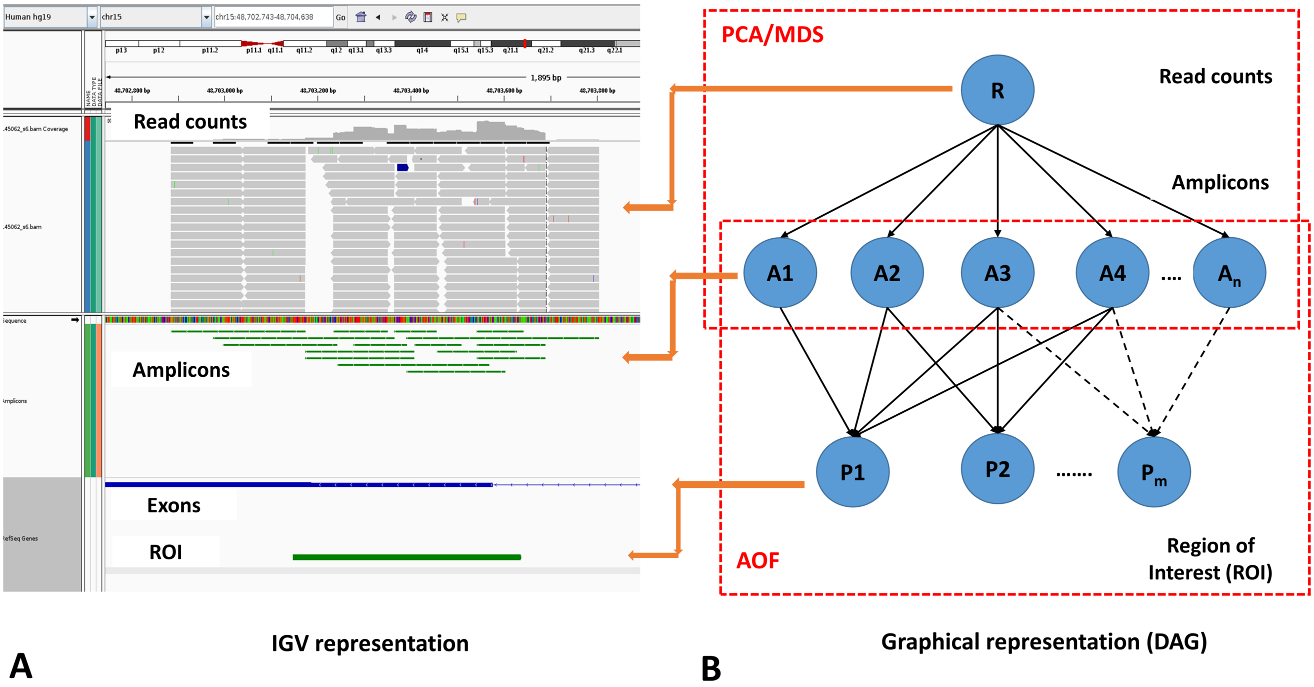

Figure S1.1: Flow of information in amplicon sequencing data. A) Example depth of coverage representation for targeted exonic region of FBN1 gene plotted using IGVTools. The top of panel shows the reads that are aligned to the position encoded by the amplicons. The green horizontal dashed lines in the middle are overlapping structure of the amplicons. Finally, in the bottom represents the targeted exonic region represented by thick green horizontal bar. **B**) The flow information in panel A is encoded by the graphical representation (directed acyclic graph) encapturing the various dependencies between the three panels. The nodes of this graphical structure are Read counts, Amplicons and Positions or the targeted region.

S2 GC content correction

GC content correction is done by loess based linear regression method to account for biases introduced by GC rich region in the target region. In order to measure the effect of GC content on the read count data it is important to know how much of these fraction correlate with the read counts for all the target regions. Figure S2.1 and Figure S2.2 shows the correlation between these two entities computed for each samples across all 5 batch sets of TAAD panel divided into autosomal and sex chromosomal targets.

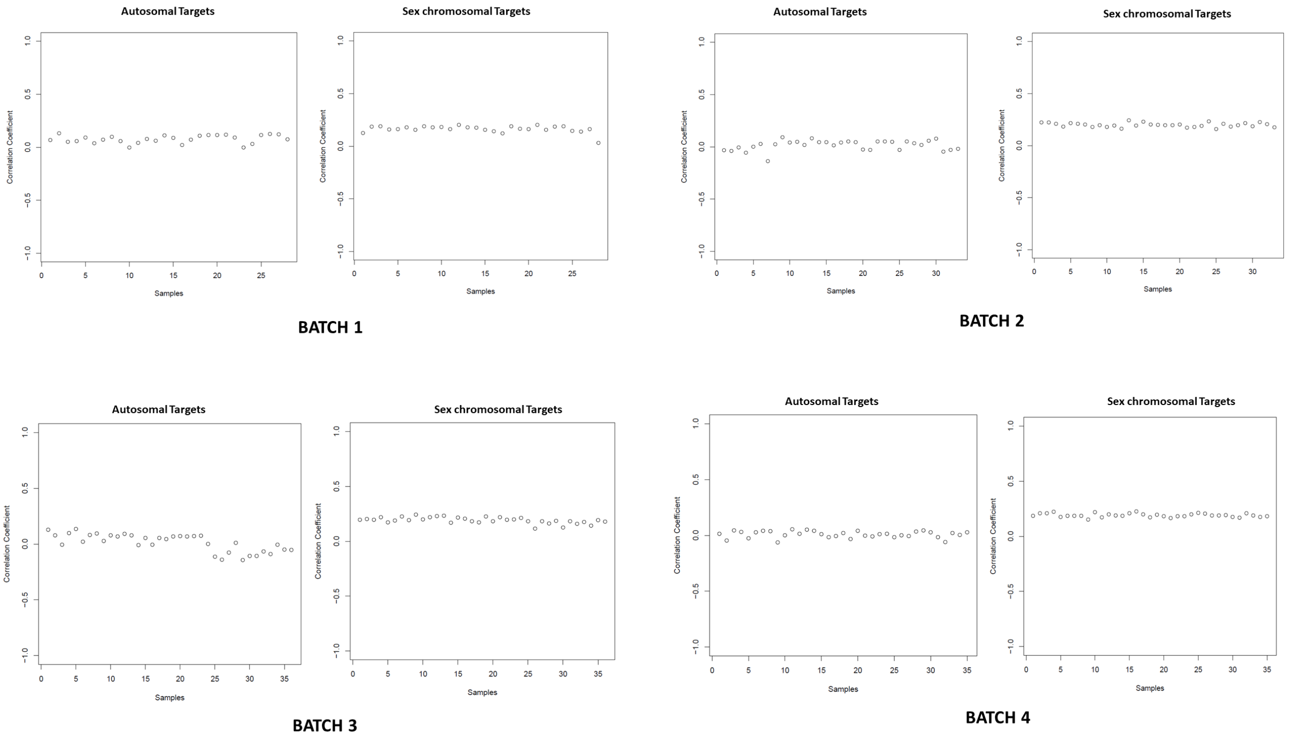

Figure S2.1: Correlation plot of GC content fraction with target read counts. For samples of each of the batches 1-4 the GC fraction computed for each of the target amplicons were correlated with their respective read counts. The correlation plot was obtained separately for autosomal and sex chromosomal targets.

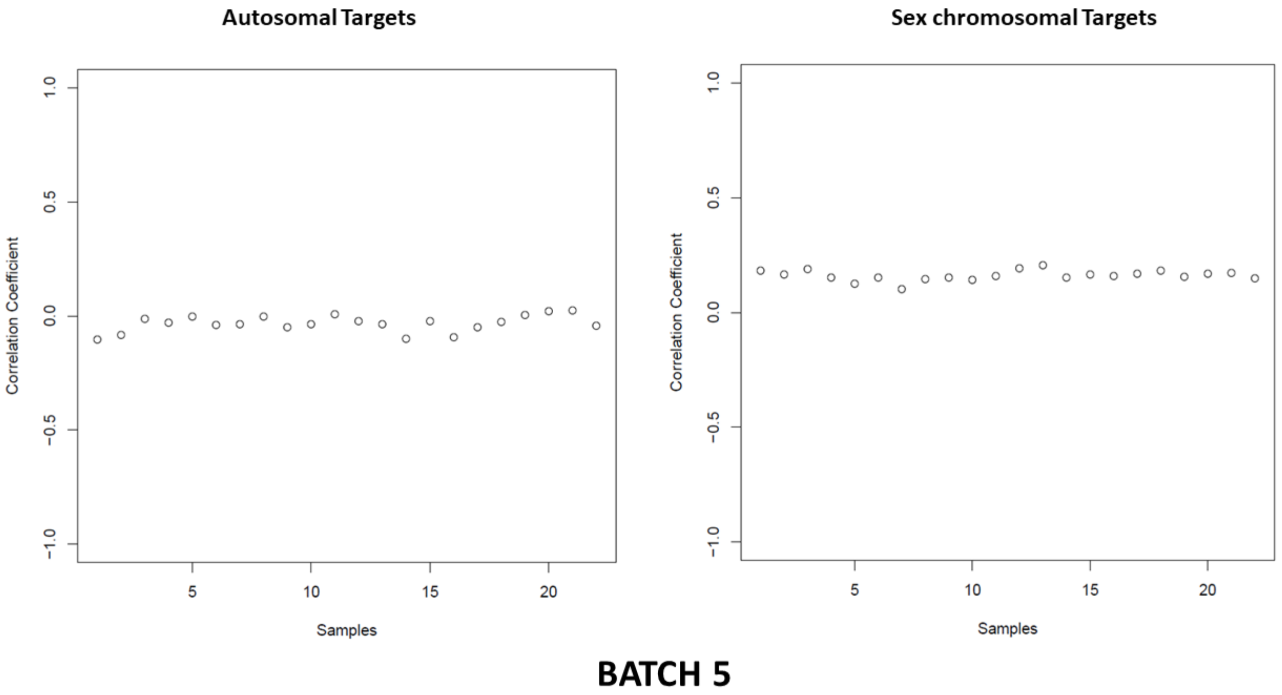

Figure S2.2: Correlation plot of GC content fraction with target read counts. For samples of the batch 5, the GC fraction computed for each of the target amplicons were correlated with their respective read counts. The correlation plot was obtained separately for autosomal and sex chromosomal targets.

S3 Controlling the variance using PCA/MDS

In section S1, information flow from read counts to the targeted exonic regions (TERs) mediated via amplicons is representatively explained by Figure S1.1. Primarily, From theoretical perspective, the goal is to handle the uneven variation present in the data. Hence, the ideal way to deal with such situation is to explore the variation present in the amplicon read counts (RC) within the sample and across the samples. This can be achieved using principles of principal component analysis (PCA).

Intuitively, the raw read counts of all the samples per batch is stored in as amplicon-by-sample RC data matrix whose rows are represented by amplicons and columns by sample names. It is normalized for GC content and mean within samples. Variability of normalized RC of amplicons can be understood by computing the sample covariance matrix of amplicon RC.

Variance of any random variable x_1 is defined as:

$$var(x_1) = E\{x_1^2\} - (E\{x_1\}^2) \tag{1}$$

This can also be written as $var(x_1) = E\{(x_1^2 - E\{x_1\}^2)\}$, which clearly shows that variance measures the average deviation from the mean value. For more than one random variable it is useful to analyze the covariances given by:

$$cov(x_1 x_2) = E\{x_1 x_2\} - E\{x_1\}E\{x_2\} \tag{2}$$

For our Amplicon-by-Sample Read depth count data matrix, the major goal is to understand the variability of the read depth of amplicons within the sample and across the sample. Specifically, we want to know the structure of the variance of the amplicons in the data. This can be better understood by computing the sample covariance matrix of amplicons. If we assume $x_1, x_2.., x_n$ as set of our amplicons then

$$\mathbf{C(x)} = \begin{bmatrix} cov(x_1, x_1) & cov(x_1 x_2) & cov(x_1 x_3) & \dots & cov(x_1 x_n) \\ cov(x_2, x_1) & cov(x_2 x_2) & cov(x_2 x_3) & \dots & cov(x_2 x_n) \\ \dots & \dots & \dots & \dots & \dots \\ cov(x_n, x_1) & cov(x_n x_2) & cov(x_n x_3) & \dots & cov(x_n x_n) \end{bmatrix} \tag{3}$$

By combining equation 2 and 3 and extending it as matrix notation we get:

$$\mathbf{C(x)} = E\{\mathbf{x}\mathbf{x}^T\} - E\{\mathbf{x}\}E\{\mathbf{x}\}^T \tag{4}$$

Eventually, if the variables are uncorrelated, the covariance matrix is diagonal which means they are correlated to themselves. If they are all further standardized to unit variance then covariance matrix is identity matrix.

S3.1 Eigen Value decomposition of Covariance matrix is basic PCA analysis

Maximization and minimization of variance of any linear combination of random variable can be computed by optimization of the covariance matrix of the data. Consider any linear combination $w^T x = \sum_i w_i x_i$ we can compute its varaince simply by:

$$E\{(\mathbf{w}^T\mathbf{x})^2\} = E\{(\mathbf{w}^T\mathbf{x})(\mathbf{x}^T\mathbf{w})\} = E\{\mathbf{w}^T(\mathbf{x}\mathbf{x}^T)\mathbf{w}\} = \mathbf{w}^T E\{\mathbf{x}\mathbf{x}^T\}\mathbf{w} = \mathbf{w}^T\mathbf{C}\mathbf{w} \tag{5}$$

Assuming mean is zero hence $E\{x\} = 0$. From equation 4 we can see that basic problem of PCA can be seen as optimizing Covaraince matrix $E\{\mathbf{x}\mathbf{x}^T\}$ for some optimal weight vectors $\mathbf{w}$. From linear algebra we can see that the covariance matrix C can be decomposed as:

$$\mathbf{C} = \mathbf{U}\mathbf{D}\mathbf{U}^T \tag{6}$$

where U is an orthogonal matrix, and $\mathbf{D} = diag(\lambda_1, ..., \lambda_m)$ is diagonal. The columns of U are the *eigenvectors* of $\mathbf{C}$, and the λ_i are the corresponding *eigenvalues.*

All the principal components can be found by ordering eigenvectors $\mathbf{u}_i$, $i = 1, ...m$ in $\mathbf{U}$ so that the corresponding eigenvalues are in decreasing order. If $\mathbf{U}$ is ordered then the i-th principal component $\mathbf{s}_i$ is equal to:

$$\mathbf{s}_i = \mathbf{u}_i^T\mathbf{x} \tag{7}$$

Now, the columns of the matrix $\mathbf{U}$ are arranged according to corresponding eigen values hence these are the directions that explain maximum variance present in the data.

For our normalized amplicon RC data matrix, the aim is to reduce the variance. Hence we remove first k columns of matrix $\mathbf{U}$ by projecting the input data matrix to the reduced column matrix of U. The reduced column matrix of eigen vectors is given by:

$$\mathbf{A}_{n\times k} = \mathbf{U}_{n\times k} \tag{8}$$

Finally, in order to get the denoised original amplicon-by-sample matrix we do this by:

$$\hat{\mathbf{X}} = AA^T X \tag{9}$$

Here, the matrix A is obtained from equation 8. In the current analysis we aim to remove approximately 80% of the variance present in the data. Hence, choosing first k columns of the eigen vector we double re-project the unit-norm and zero mean centered data on to it. This denoised matrix is finally used for computing Log_2R ratios and subsequent segmentation using CBS algorithm.

S3.2 Using MDS gives identical result as PCA

One limiting factor for performing PCA is computing the covariance matrix XX^T and subsequent EVD step. Conventionally, we represent this matrix as read count matrix whose rows are amplicons and columns as samples. Analyzing large gene panels involve large set of amplicons and thus computing of covariance matrix and EVD becomes computational intensive. Hence, we implemented metric multi-dimensional scaling (MDS) approach to address this issue by using euclidean distance measure between the data points. MDS and PCA are connected as they both address towards solving of $x^T x$ or xx^T matrix. Euclidean distance measure of the amplicon read count matrix is given by:

$$d_{ij} = \text{distance between data points } x_i \text{ and } x_j. \tag{10}$$

If we denote each data point as $\mathbf{x}_i$ then euclidean distance between them is given by:

$$d_{ij} = ||x_i - x_j||^2 = ||x_i||^2 + ||x_j||^2 - 2x_i^T x_j \tag{11}$$

If we now normalize these distance such that each row and column sum is zero then we obtain matrix $X^T X$ multiplied with some constant. EVD of this matrix is same as that of covariance matrix XX^T. The only difference is that in this case the EVD is done on the column side of the matrix $(X^T X)$. If we recall the amplicon-by-sample read count matrix the rows are amplicons and columns are samples, then this representation using MDS approach reduces the computational complexity. Hence, we obtain identical result as that of PCA. Finally, once the eigen vectors have been computed then we perform same step as in equation 9 for denoisation of the read count matrix.

S3.3 Distribution of Log_2R score across samples

Figure S4.1 represents the distribution of Log_2R score per amplicon across all the samples for BATCH3 of the TAAD panel. This batch has 5 TPs: deletions (T3,T5,T6,T8) and 1 duplication (T4). The Log_2R score is computed using leave-one-out approach where set of reference samples are selected among all other samples except the target sample as described in Section 2.2.5 in the manuscript.

When the read count (RC) is normalized for mean coverage and corrected for GC content without applying PCA/MDS denoising the distribution of Log_2R score is represented by Figure S4.1A. On the other hand Figure S4.1B gives the distribution of Log_2R score when the normlaized RC is denoised using PCA/MDS approach. It can be seen that because of the denoising step the overall noise gets reduced in Figure S4.1B. Also, the signal pertaining to deletion & amplification can be seen enhanced around the amplicon numbered 2800 - 3500 (700 amplicons). The zoomed in version of the distribution plot for these set of amplicons is represented by Figure S4.1C.

These amplicon region have an overlap with *FBN1* gene. From Supplementary File 1: sheet 6, it can be deduced that possibly these signals approxiamtely correspond to 5 deletions (peaks marked by overlapping colors of purple,brown etc) and 1 duplication (peaks marked by oceanic-blue color)

signal. Additionally, this distribution plot explain the reason change point event detection algorithm such as CBS is able to correctly predict these as CNV regions as none of other neighbourhood regions has such distinguished peaks.

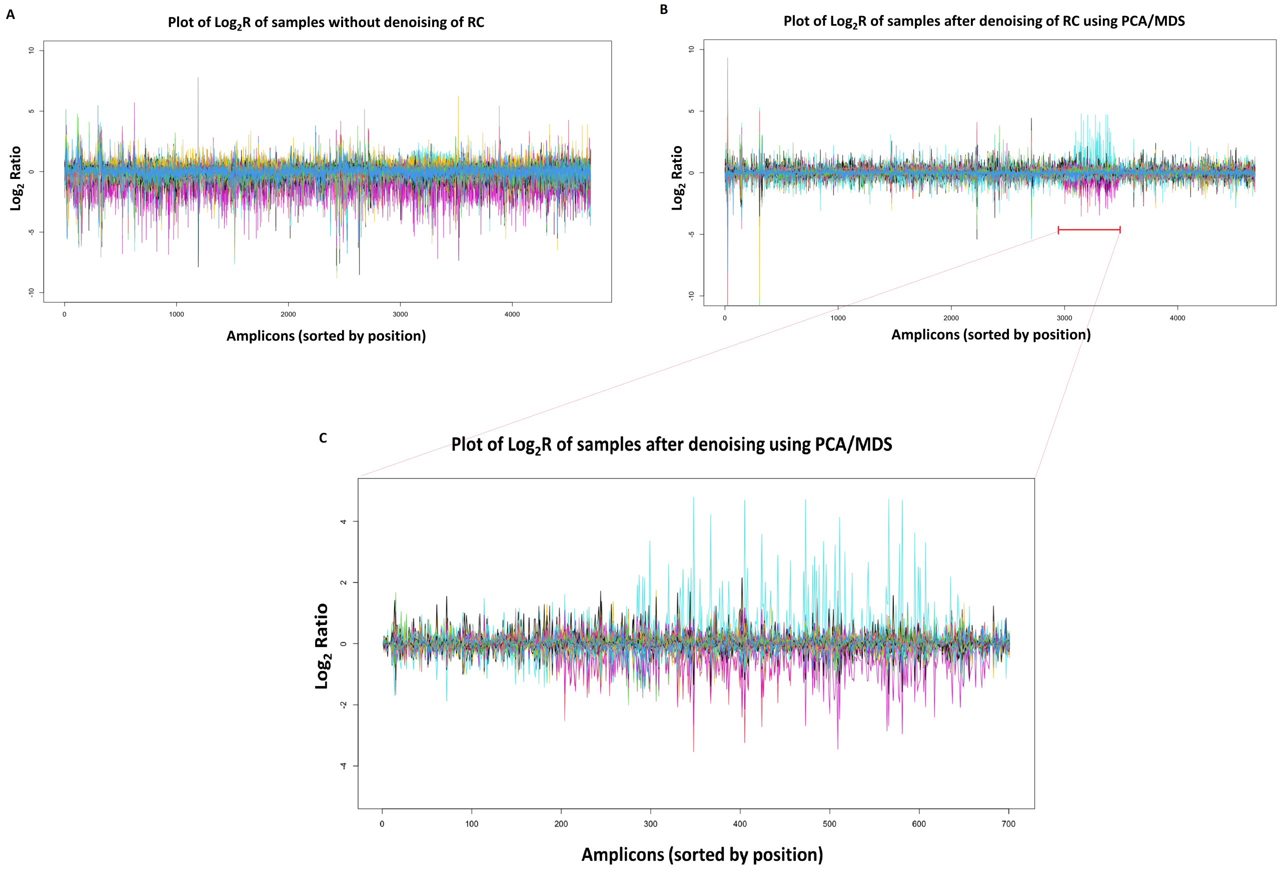

Figure S3.1: Plot of Log_2R score per amplicon across all the samples of BATCH 3. The X-axis represents the amplicons sorted according to position. The Y-axis denote the Log_2R score obtained by leave-one-out approach. **A**) Represents the distribution of the Log_2R per amplicon across all the samples when read count (RC) is only normalized by mean and GC corrected. **B**) Represents the distribution of the Log_2R score when normalized RC is denoised using PCA/MDS approach and **C**) Represents the zoomed region of 700 amplicons that span the *FBN1* gene. It can be observed distinct continuous signal of deletions (-ve log_2R purple peaks) and amplification (+ve log_2R oceanic blue peaks) score as compared to neighbourhood peaks.

S4 Computing AOF

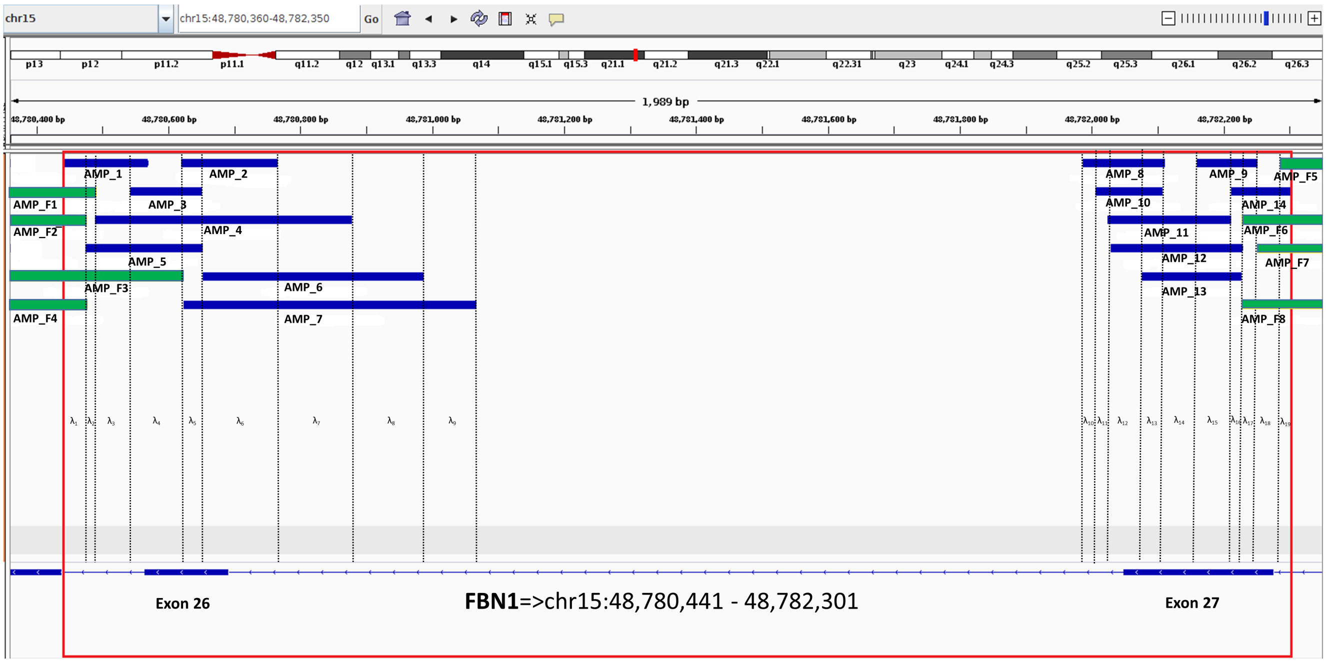

Figure S4.1: Understanding amplicon overlap filtering (AOF) approach. A) An IGVtools representation of CNV segment chr15:48780441-48782301 with amplicons encompassing this region where two exons (exon 26 and exon 27) were targeted. The red colour rectangular box represent the boundary of the CNV region. **Overlapping amplicons retrieval**: the blue horizontal bars represents the amplicons that were utilized by the CBS algorithm in the DS approach. They all lie within the CNV region starting from AMP_1 to AMP_14. The green horizontal bars are the amplicons that flank or overlap the predicted CNV region but are not utilized by CBS algorithm for prediction. **Partitioning the CNV segment:** Aligned amplicons (*flanking* and *within*) are partitioned according to their start and end position. Each partition has fixed width or equal length of amplicons. The black vertical dotted line denotes the partition. **Averaging *Log*2R across partitions:** For any given partitioned segment, the *Log*2R ratio is averaged proportionally with respect to width of partition segment.

We described two approaches for predicting CNV segments: (1) using direct segmentation (DS) and (2) using amplicon overlap filtering (DS-AOF) approach. According to the Figure S1.1 the flow of information in the amplicon sequencing data as represented using graphical model shows the dependency structure of amplicons with respect to the genomic position (upper half of the graph).

The aim of DS-AOF approach is to utilize this dependency structure to average out the Log_2R ratios of each of these amplicons. The DS-AOF approach is applied to filter out the potential false positive segments. It works in three stages as shown in Figure S4.1 for an example CNV segment predicted by DS approach in Batch 3 (see supplementary file 1: sheet 3). The CNV segment is of FBN1 gene having coordinates as chr15:48780441-48782301 encompassed by 14 amplicons.

- **Overlapping amplicons retrieval**: In Figure S4.1, for the given CNV segment all the 14 amplicons are retrieved as shown in blue horizontal lines starting from amplicon AMP_1 (start of the segment) and AMP_14 (end of the segment). There are eight other amplicons marked in green which either start or end segments partially overlap with the predicted CNV segment and are called as "flanking" amplicons. In the DS approach these flanking amplicons were not included for predicting the CNV segments. However according to dependency model of flow of information as shown in Figure S1.1 these flanking amplicons are related to the included amplicons because they overlap. Hence for computing the final segmental average Log_2R ratios should include the contribution from the flanking amplicons. Thus for the given predicted CNV segment all the amplicons that overlap with this region are retrieved.

- **Partitioning the segments**: After retrieving all the segments (including flanking and included amplicons) that encompass with the predicted CNV segment we partition it. The segment is partitioned according to start and and end of the amplicons such that each partition has fixed width or equal length of amplicons. An example demonstration of this partitioning step is demonstrated in Figure S4.1 where the predicted CNV segment is partitioned into 19 intervals denoted as $\lambda_1 ... \lambda_{19}$. For example, λ_1 segment starts with starting point of *within* amplicon AMP_1 marked as blue horizontal bars and ends with *flanking* amplicons (green horizontal bars) AMP_F1 and AMP_F2.

- **Averaging out the Log_2R ratios**: For each of these partitioned interval we average the Log_2R associated with each of the amplicons for a given interval. The averaged Log_2R ratio for the overall segment is given by :

$$\text{Avg. Log}_2\text{R} = \sum_{j}^{N} \frac{\sum_{i}^{M_j} \text{Log}_2\text{R}_{\text{AMP}_{ij}} \times \|\lambda_j\|}{M_j} \qquad (12)$$

where N is the total numner of partitioned interval segments, M_j is the total number of amplicons that corresponds to any given partitioned interval λ_j and $\|\lambda_j\|$ is the length of the partitioned interval segment.

As an example, for Figure S4.1 the CNV segments were paritioned into λ_j segments where $j \epsilon \{1..19\}$. For the first segment interval $j = 1$, the corresponding amplicons are AMP_1, AMP_F1, AMP_F2, AMP_F3, AMP_F4. Hence, the value of $M_j = 5$ for this partition. The length of this

interval segment $\|\lambda\|$ is given by difference between start position of AMP_1 and the end position of AMP_F4. Thus the corresponding $\text{Log}_2\text{R}_{\lambda_{j=1}}$ ratio for λ_1 segment is:

$$\text{Log}_2\text{R}_{\lambda_{j=1}} = \frac{(\text{Log}_2\text{R}_{AMP\_1j} + \text{Log}_2\text{R}_{AMP\_F1j} + \text{Log}_2\text{R}_{AMP\_F2j} + \text{Log}_2\text{R}_{AMP\_F3j} + \text{Log}_2\text{R}_{AMP\_F4j}) \times \|\lambda_j\|}{M_j = 5} \tag{13}$$

For seconda segment when $j = 2$, the length of the partitioned interval $\|\lambda_2\|$ segment is given by difference between start position of AMP_5 and end position of AMP_F1. The corresponding $\text{Log}_2\text{R}_{\lambda_{j=2}}$ ratio is:

$$\text{Log}_2\text{R}_{\lambda_{j=2}} = \frac{(\text{Log}_2\text{R}_{AMP\_1j} + \text{Log}_2\text{R}_{AMP\_F1j} + \text{Log}_2\text{R}_{AMP\_F5j} + \text{Log}_2\text{R}_{AMP\_F3j}) \times \|\lambda_j\|}{M_j = 4} \tag{14}$$

Similarly, for the last segment when $j = N$ where N=19. The length of the partitioned interval $\|\lambda_{19}\|$ segment is given by difference between start positon of AMP_F5 and the end position of AMP_14. The corresponding $\text{Log}_2\text{R}_{\lambda_{j=19}}$ ratio is:

$$\text{Log}_2\text{R}_{\lambda_{j=2}} = \frac{(\text{Log}_2\text{R}_{AMP\_F5j} + \text{Log}_2\text{R}_{AMP\_14j} + \text{Log}_2\text{R}_{AMP\_F6j} + \text{Log}_2\text{R}_{AMP\_F7j} + \text{Log}_2\text{R}_{AMP\_F8j}) \times \|\lambda_j\|}{M_j = 5} \tag{15}$$

Finally, the average Log_2R score of the CNV segment is given by summing up of all the respective $Log_2\text{R}$ ratios of these partitioned segments given by:

$$\text{Avg. Log}_2 R = \sum_{j=1}^{N=19} \text{Log}_2 R_{\lambda_j} \tag{16}$$

From equation 12 or 16 we obtain the new averaged $Log_2\text{R}$ ratio score for the predicted segments. Thus application of amplicon overlap filtering (AOF) helps in providing realistic estimate of the segment means as compared to those obtained by using direct segmentation (DS) approach by using CBS algorithm. Together we define this strategy for filtering out the segments as DS-AOF.

S4.1 Application of DS-AOF

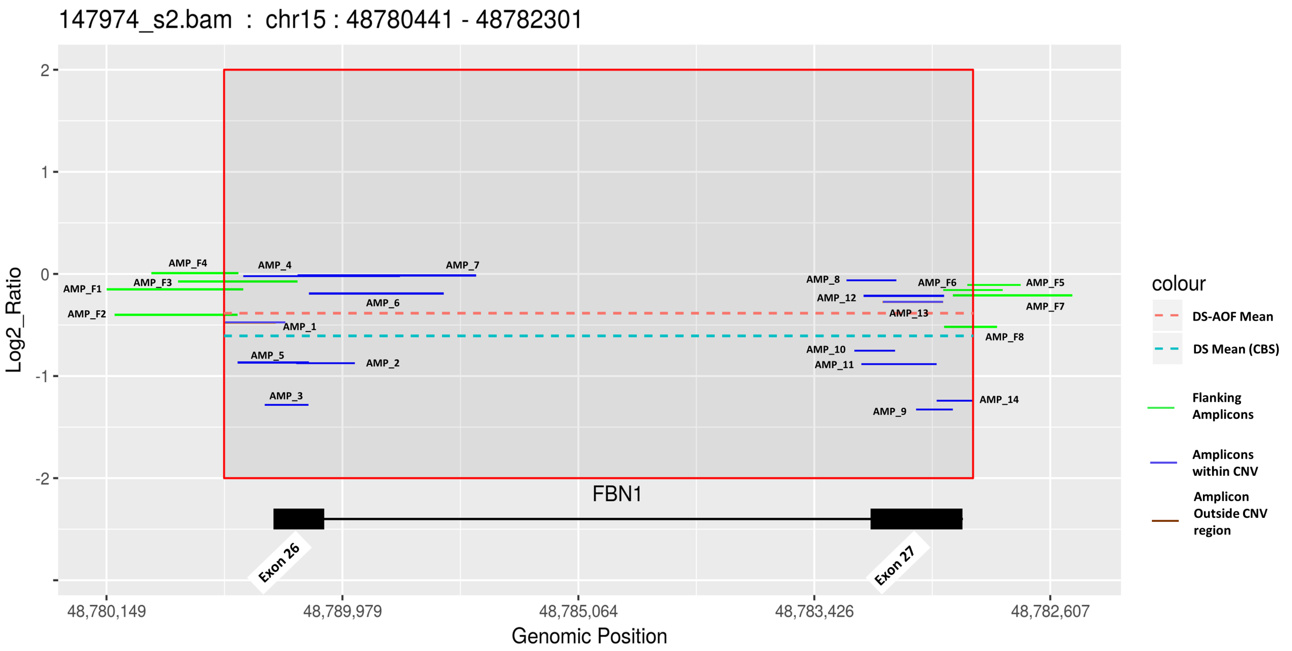

Figure S4.2: Visualization of an example CNV segment using DS-AOF approach The CNV segment corresponds to *FBN1* gene having coordinates as chr15:48780441-4878230 enclosed in red box. The X-axis represent the positions of the respective exons and the Y-axis represent the actual Log_2R ratio values of amplicons obtained after PCA/MDS based normalization. The amplicons are plotted as horizontal lines (blue/green) according to their genomic coordinates. Amplicons that are marked as blue are the ones that led to the generation of this particular CNV segment using the CBS algorithm. And the green amplicons correspond to those amplicons that did not participate in CNV segment generation but overlaps with this CNV segment. The dotted lines marked in as light-blue and orange colour represent the average segmental Log_2 R scores obtained using DS-approach or CBS algorithm and DS-AOF approach respectively.

We demonstrate the application of DS-AOF approach in filtering out the CNV segment (*FBN1* gene; chr15:48780441-48782301) obtained using DS approach as shown in Figure S4.2. Here the amplicons are plotted according to their Log_2R scores having similar intuitive representation as shown in Figure S4.1. We can see that the 14 amplicons that led DS-based approach to predict as potential CNV segment are marked with blue horizontal lines. These amplicons result in segmental average Log_2R score of -0.61 (blue dotted line) which is less than the segmentation threshold (ST) for deletion (≤ -0.50) thereby making a mis-classification.

With incorporation of Log_2R score values from the *flanking* amplicons and combining their

overlap dependencies with the 14 amplicons results in the segmental average Log_2R score of -0.3163 (orange dashed line). This value is higher than the ST value of (≤ -0.50) and thus helps in classifying this CNV segment as a false positive. Eventually, this makes DS-AOF strategy a useful approach in filtering out the false positives (FPs).

S4.1.1 Filtering via annotation and visualization

The predictive power of varAmpliCNV from the Haloplex based TR data ranges from single to integer number of exon deletion/duplication. We exclude any CNVs that are partially deleted/duplicated of the targeted exonic region(s). Filtering such segmental CNVs can be challenging through DS-AOF approach. The primary reason being that it only provides quantitative score of the underlying CNV via the number of amplicons encompassing the targeted region. It incorporates amplicons that lie both *within* and *flanking* the CNV region boundary (blue and green amplicons respectively).

However, besides *within* and *flanking* amplicons there are other set of amplicons that are mapped to the targeted exonic region(s) but are not utilized by DS-AOF for predicting CNVs. We define such amplicons as *outside* and are marked in brown colour. These sets of amplicons could provide additional about true structural representation of the CNV segments. Other factors which includes length of the TERs, size of predicted CNV region in proportion to TER length and the density of the amplicons within CNV region can also help in evaluating these CNV segments.

Hence, it is necessary to annotate the predicted CNV segments. By default, varAmpliCNV annotates the segments using amplicon design file and targeted region file (with information of gene names) provided by the user. For a given gene, the TER are incrementally annotated as *Region_0, Region_1 .. Region_n.* All of these annotation and above propositions are embedded in the visualization plots that describes the TER, predicted CNV region (red colour rectangular box) and amplicons within(blue), flanking (green) and outside (brown) that span the CNV region. Finally, any computationally predicted CNV segment should be further validated by wetlab based orthogonal methods such as arrayCGH, MLPA and MAQ assay protocols for classifying them as false positive (FP) or true positive (TP). Although, it is not feasible to validate each of these predicted segments but through visualization plots the list can be trimmed down.

In subsequent section we demonstrate utility of visualization plot in filtering out the FP in TAAD panel. Based on this we derive set of rules that can be generalized while using visualization plot and are then applied to the deafness panel dataset for filtering out FPs.

S4.1.2 TAAD panel

Figure 2B in the main manuscript, presents 11 CNV segments (TP:9 and FP:2) that were obtained using DS-AOF approach and were validated using orthogonal methods. Figure S4.3 to Figure S4.13 are the corresponding visualization plots of these 11 CNV segment region annotated with genes and all the set of amplicons aligned to respective TERs.

For the data point T14 from BATCH-4, Figure S4.12 corresponds to the visualization plot and has same generalized representative description as of Figure S4.2. It shows duplication of TER *Region_0* of the *FOXE3* gene. Specifically, it can be observed that the predicted CNV region (red

box) partially overlaps with *Region_0.* The density of amplicons within (maked blue) and flanking (marked green) is high. Also, a small number of outside amplicons (marked brown) are aligned to other part of *Region_0.* It was found (Supplementary data: sheet 6) that the datapoint T14 is a FP. Could this be classified as FP just by using visualization plot only without using any experimental validation?

The answer is primarily is yes because the TR based NGS data utilizes read depth methodology (RD) to detect CNVs and its resolution is limited to single or integral number of TERs. Finding any partial deletion/duplication of such TERs is difficult and might require additional strategy such as paired end read mapping support. Hence, such partial segmental deletion/duplication cannot be real and might be an artifact arising due to selective set of amplicons. Ideally, all amplicons should fall within or be flanking the CNV region for calling a deletion/duplication. Figure S4.12 exemplifies our reasoning that although data point T14 passes our ST segmentation threshold but its CNV status (single exon duplication) could not be replicated by orthogonal methods owing to fact that it is partially duplicated.

In case of 9 TPs as shown in Figure S4.3 - S4.8, Figure S4.9, S4.11 and S4.13 it can be uniformly seen that CNV regions completely overlaps with TERs. None of these CNV segments had any partial deletion/duplication of TERs. Amplicons encompassing the TERs were primarily within and flanking i.e non of the outside brown coloured amplicons were observed.

Figure S4.9 corresponds to visualization of data point T11 in BATCH-4. It has CNV in three TERs (*Region_1, Region_2 and Region_3*) of *COL3A1* gene. The CNV region (red box) encompasses all the three TERS. Also, the density of amplicons within and flanking all are subjected to this region and no other set of amplicons (marked brown) are aligned outside this region. In first instance, based on these plots and in combination with DS-AOF threshold value it indicates that the predicted CNV segment is a potential TP. However, upon validation by the orthogonal methods (Supplementary data: sheet 6) it turned out to be a FP. Hence, it can be further deduced that the plots seldom cannot be used to classify the CNV segments as FP or TP but should concur with findings from the orthogonal methods.

The utility of visualization plot is helpful when analyzing large panel of genes (e.g deafness panel) where application of DS-AOF approach results in large set of CNV segments as candidates for further tests that needed to be confirmed (TBC) through experimental validation by orthogonal methods.. Not all of these can be experimentally validated given the cost and manual work required. Subsequently, some of these CNV segments can be filtered using these visualization plots resulting in a trimmed down list of candidate TBC cnv lists.

In next section S4.1.3, we formulate the rules for filtering via annotation and visualization that were derived from the TAAD panel dataset. We apply these rules on deafness panel data and demonstrate its utility in blindfolded detection of TPs, pruning potential FPs and generating trimmed list of candidates for further tests that needed to be confirmed (TBC).

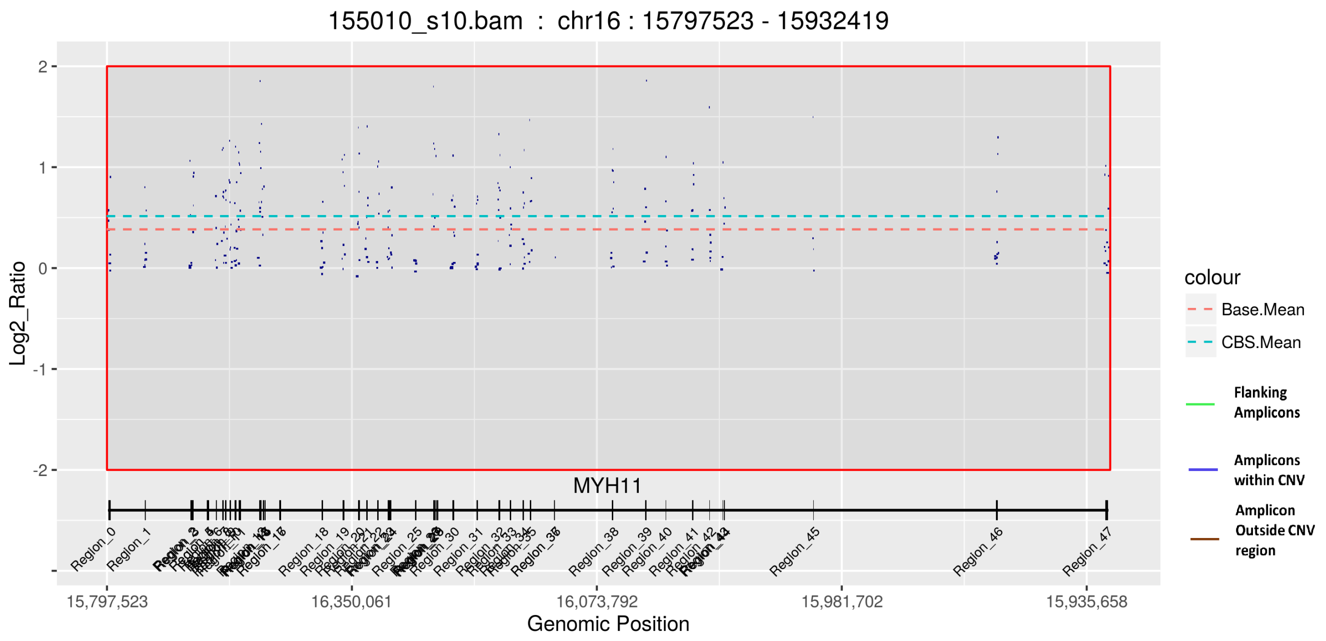

Figure S4.3: TAAD Panel-Batch1-T1: chr16-15797523-15932419-MYH11-*Duplication*. The CNV region completely overlaps with the integral number of targeted exonic regions (TER). Amplicons that are marked blue span the CNV region and TERs. None of flanking amplicons marked green and brown amplicons can be seen aligned to any other part of the CNV regions. Additionally, orthogonal methods validates the duplication of *MYH11* gene. It is classified as TP.

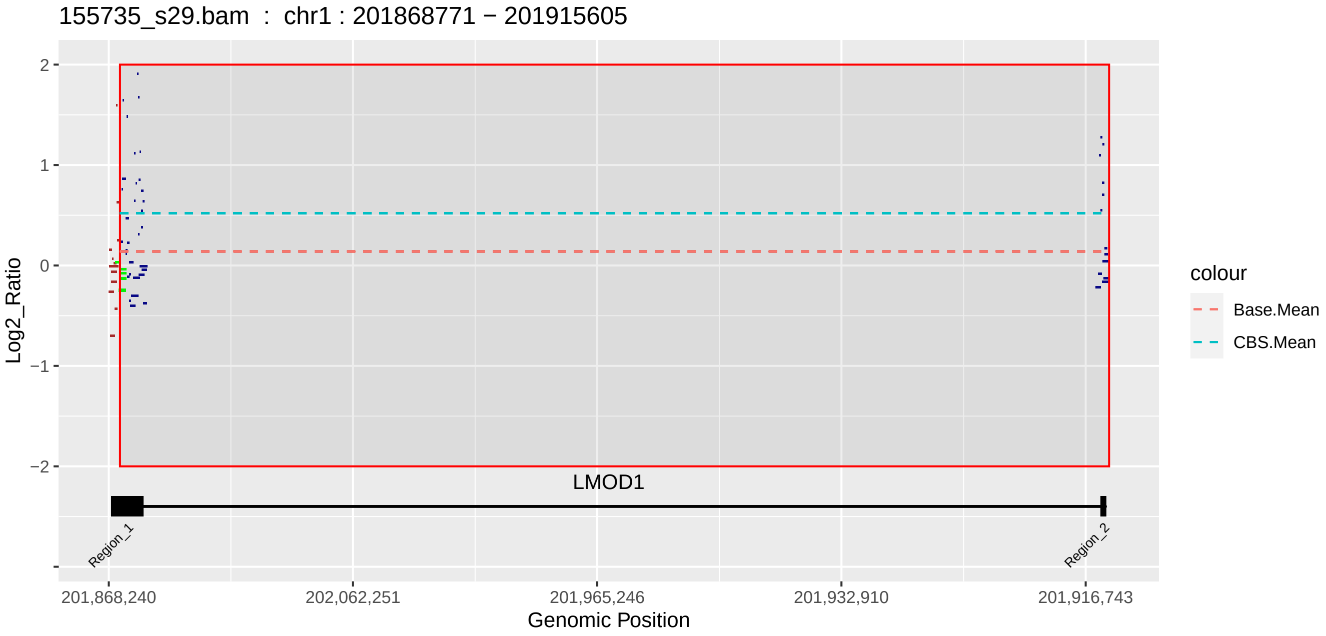

Figure S4.4: TAAD Panel-Batch1-T2: chr1-201868771-201915605-LMOD1-*Duplication*. The CNV region partially overlaps (Region_1) with the integral number of targeted exonic regions (TER). Amplicons that are marked blue span the predicted CNV region and TERs. The flanking amplicons marked green and brown amplicons can be seen aligned outside of the predicted CNV regions. Additionally, orthogonal methods donot validate the duplication of *LMOD1* gene. It is classified as FP (DS approach and k-means clustering (BIC k=2)).

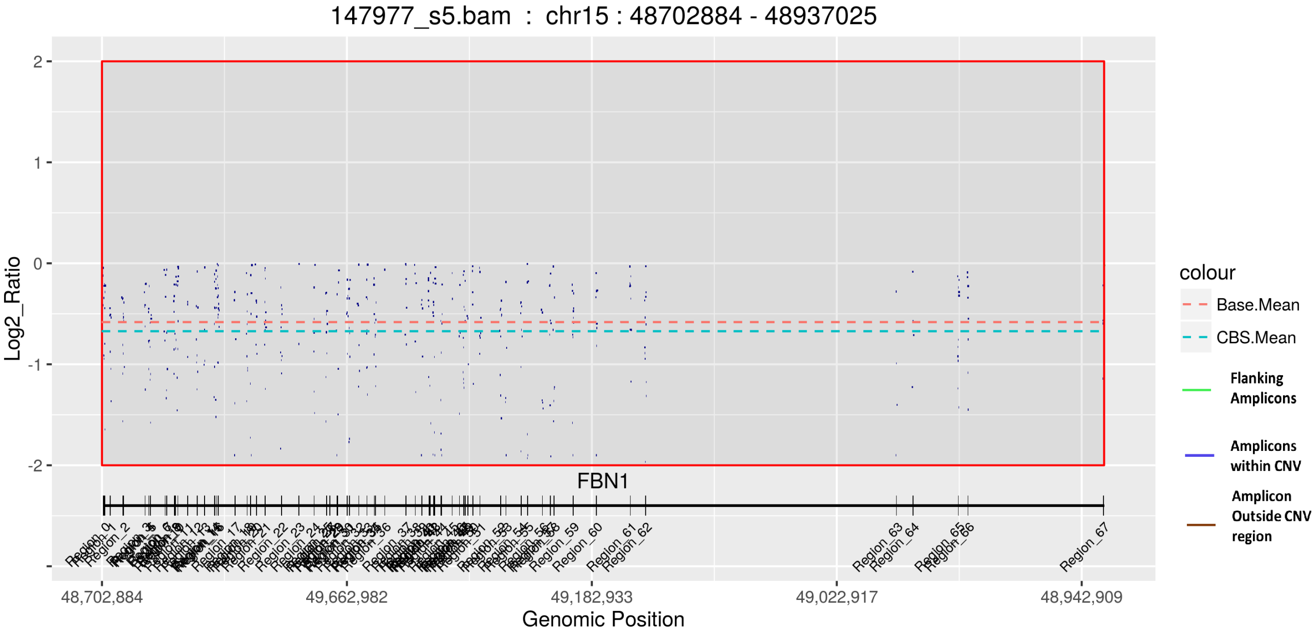

Figure S4.5: TAAD Panel-Batch3-T3: chr15-48702884-48937025-FBN1-*Deletion*. The CNV region completely overlaps with the integral number of targeted exonic regions. Amplicons that are marked blue span the predicted CNV region and TERs. None of flanking amplicons marked green and brown amplicons can be seen aligned to any part of the CNV regions. Additionally, orthogonal methods validates this as deletion of *FBN1* gene. It is classified as TP.

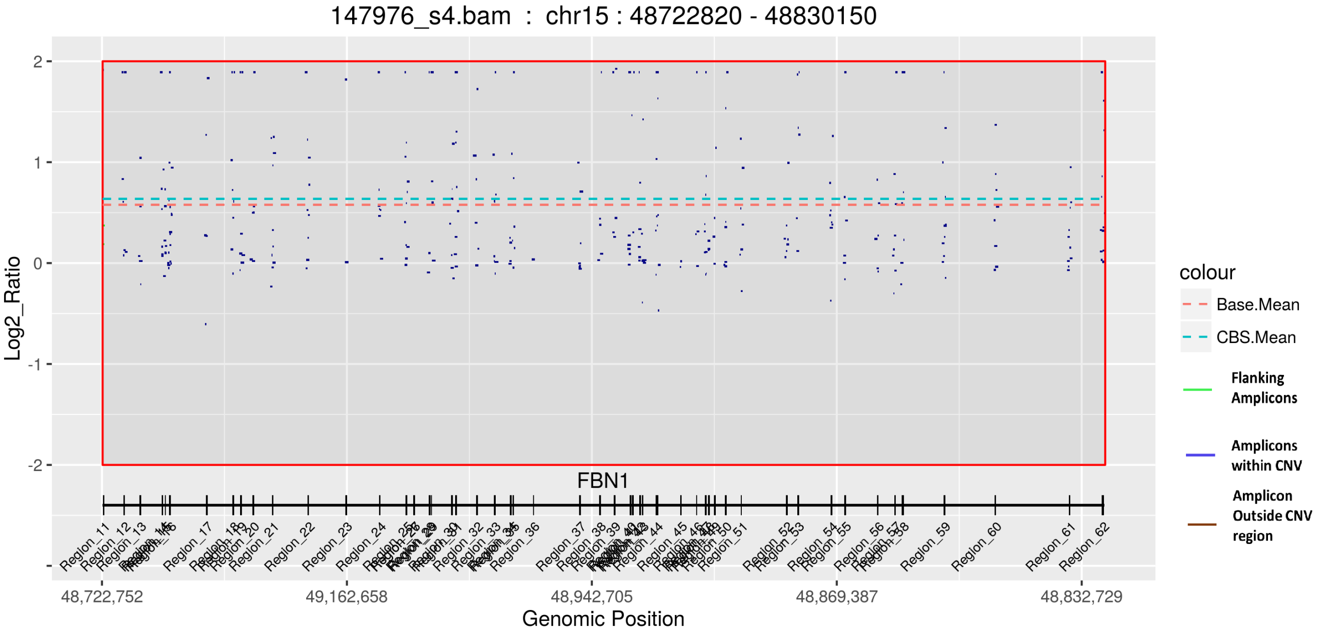

Figure S4.6: TAAD Panel-Batch3-T4: chr15-48722820-48830150-FBN1-*Duplication*. The CNV region completely overlaps with the integral number of targeted exonic regions. Amplicons that are marked blue span the predicted CNV region and TERs. None of flanking amplicons marked green and brown amplicons can be seen aligned to any part of the CNV regions. Additionally, orthogonal methods validates this as duplication of *Region_11* to *Region_62* of *FBN1* gene. It is classified as TP.

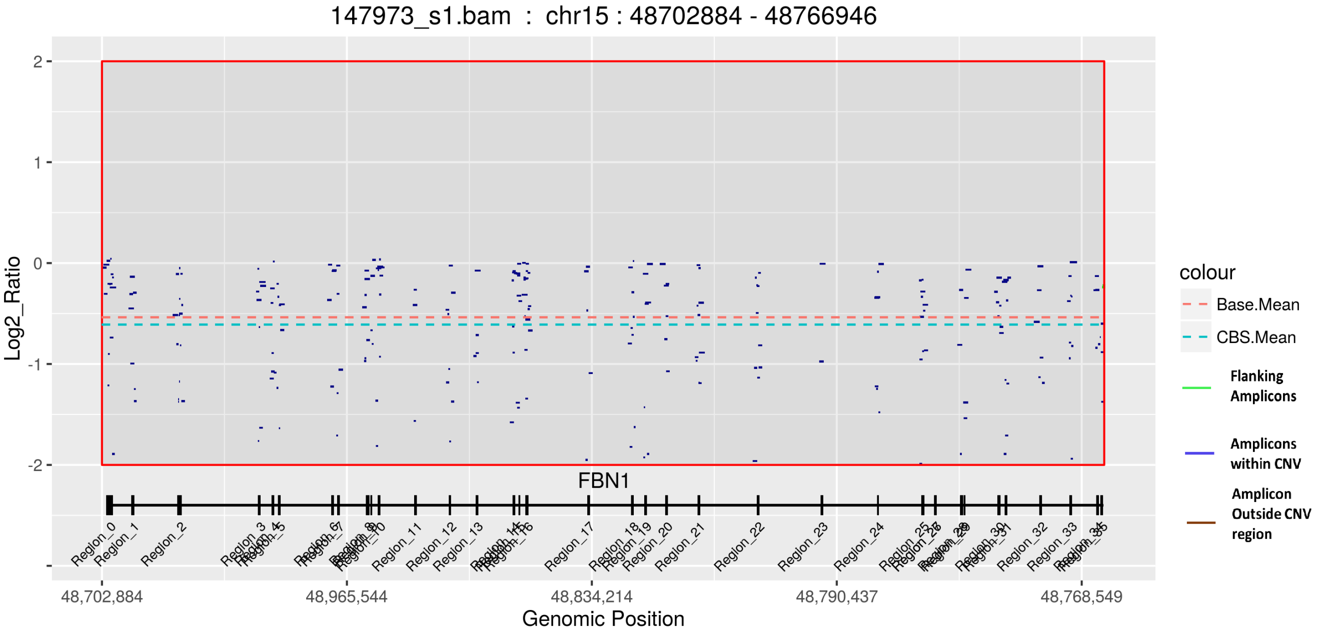

Figure S4.7: TAAD Panel-Batch3-T5: hr15-48702884-48766946-FBN1-*Deletion*. The CNV region completely overlaps with the integral number of targeted exonic regions. Amplicons that are marked blue span the predicted CNV region and TERs. None of flanking amplicons marked green and brown amplicons can be seen aligned to any part of the CNV regions. Additionally, orthogonal methods validates this as deletion of *Region_0* to *Region_35* of *FBN1* gene. It is classified as TP.

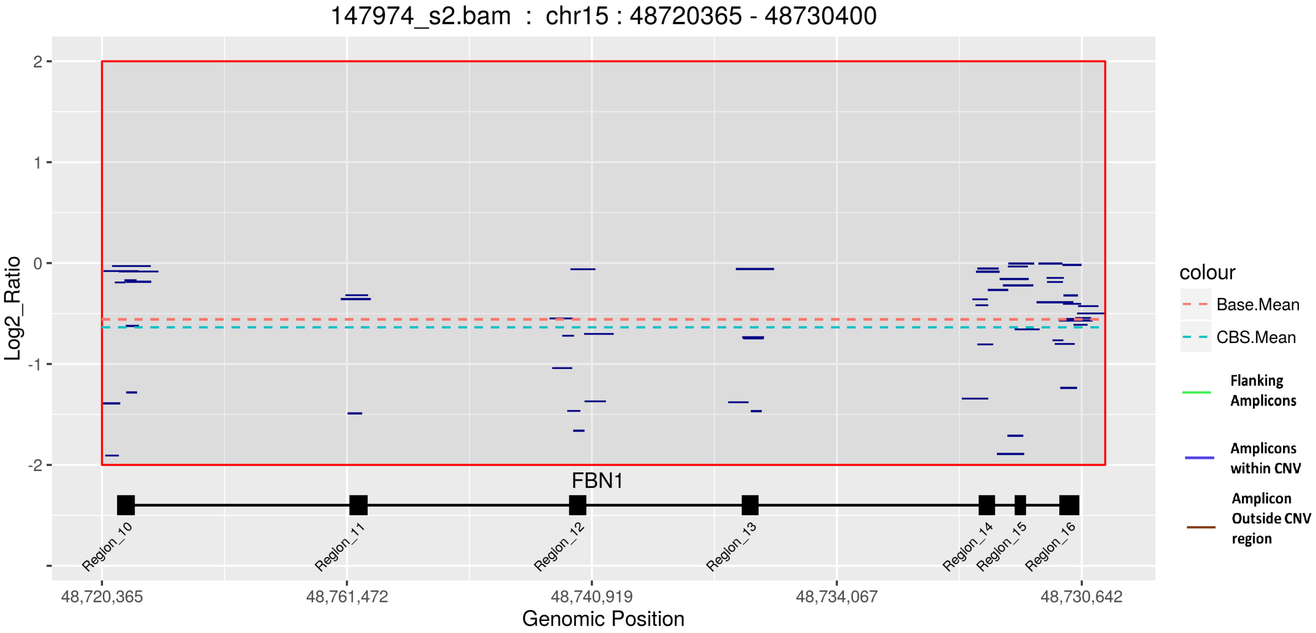

Figure S4.8: TAAD Panel-Batch3-T6: chr15-48720365-48730400-FBN1-*Deletion*. The CNV region completely overlaps with the integral number of targeted exonic regions. Amplicons that are marked blue span the predicted CNV region and TERs. None of flanking amplicons marked green and brown amplicons can be seen aligned to any part of the CNV regions. Additionally, orthogonal methods validates this as deletion of *Region_10* to *Region_16* of *FBN1* gene. It is classified as TP.

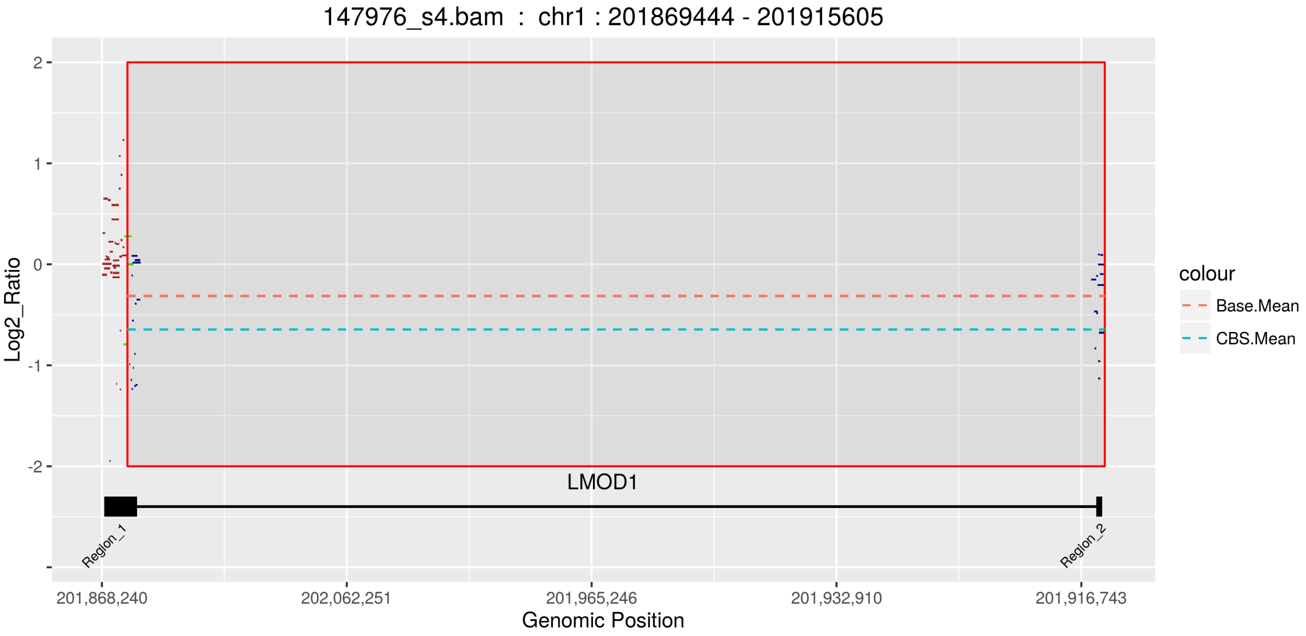

Figure S4.9: TAAD Panel-Batch3-T7: chr1-201869444-201915605-LMOD1-*Deletion*. The CNV region partially overlaps (Region_1) with the integral number of targeted exonic regions. Amplicons that are marked blue span the predicted CNV region and TERs. The flanking amplicons marked green and brown amplicons can be seen aligned to other part of the predicted CNV regions. Additionally, orthogonal methods do not validate this as deletion of *Region_1* & *Region_2* of *LMOD1* gene. It is classified as FP (DS approach and k-means clustering (BIC k=2)).

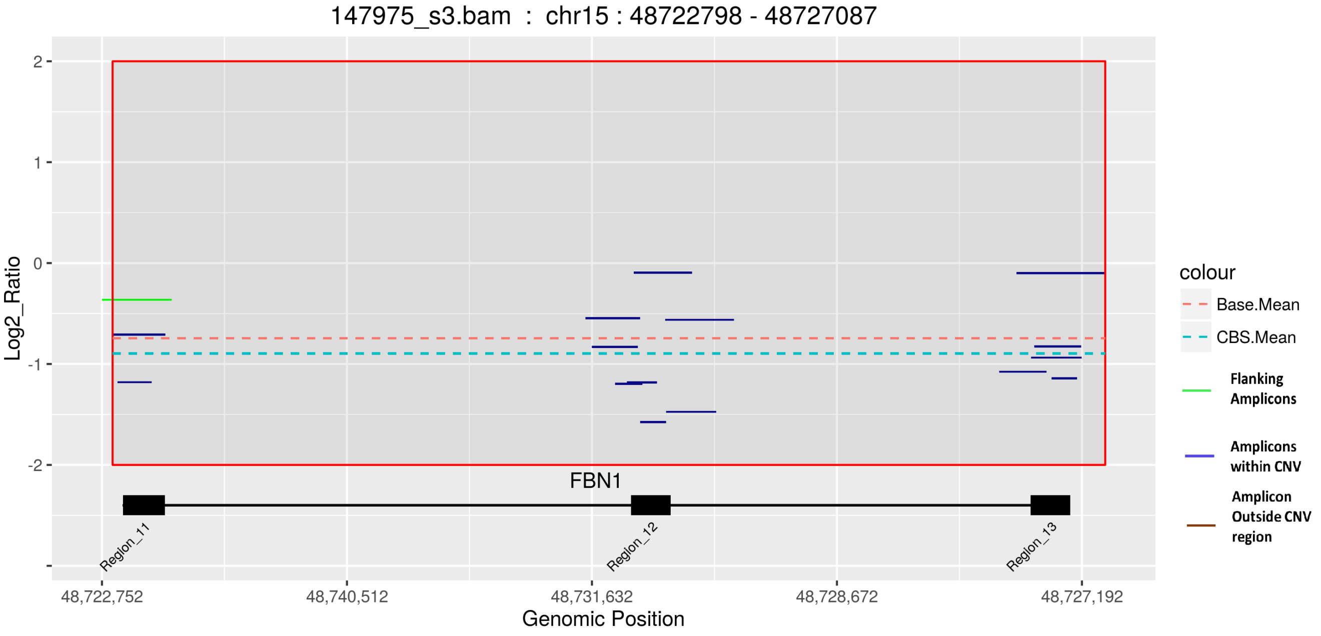

Figure S4.10: TAAD Panel-Batch3-T8: chr15-48722798-48727087-FBN1-*Deletion*. The CNV region completely overlaps with the integral number of targeted exonic regions. Amplicons that are marked blue span the predicted CNV region and TERs. None of flanking amplicons marked green and brown amplicons can be seen aligned to any part of the CNV regions. Additionally, orthogonal methods validates this as deletion of *Region_11* to *Region_13* of *FBN1* gene. It is classified as TP.

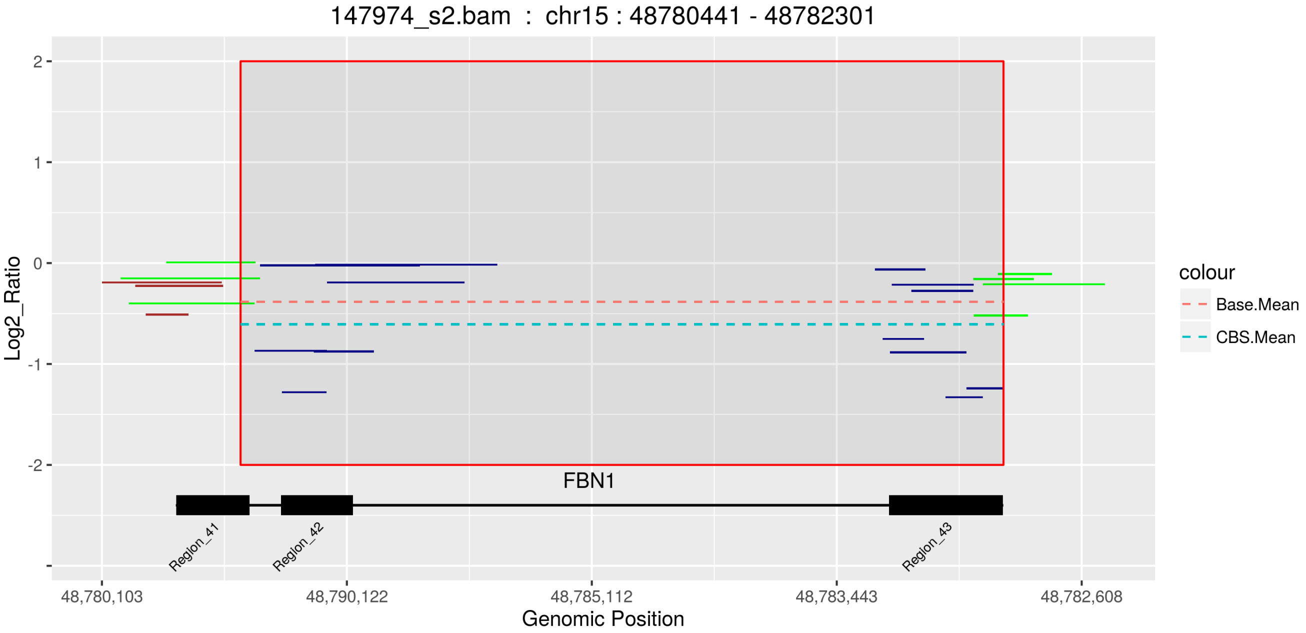

Figure S4.11: TAAD Panel-Batch3-T9: chr15-48780441-48782301-FBN1-*Deletion*. The CNV region partially overlaps (Regio_41) with the integral number of targeted exonic regions. Amplicons that are flanking (marked green) and within (marked blue) span the CNV region. Amplicons marked brown can be seen aligned to other part of the CNV region. Orthogonal methods do not validate the deletion of this CNV region (as deletion from *Region_41* to *Region_43* of the *FBN1* gene). Hence, it is classified as FP (DS approach and k-means clustering (BIC k=2)).

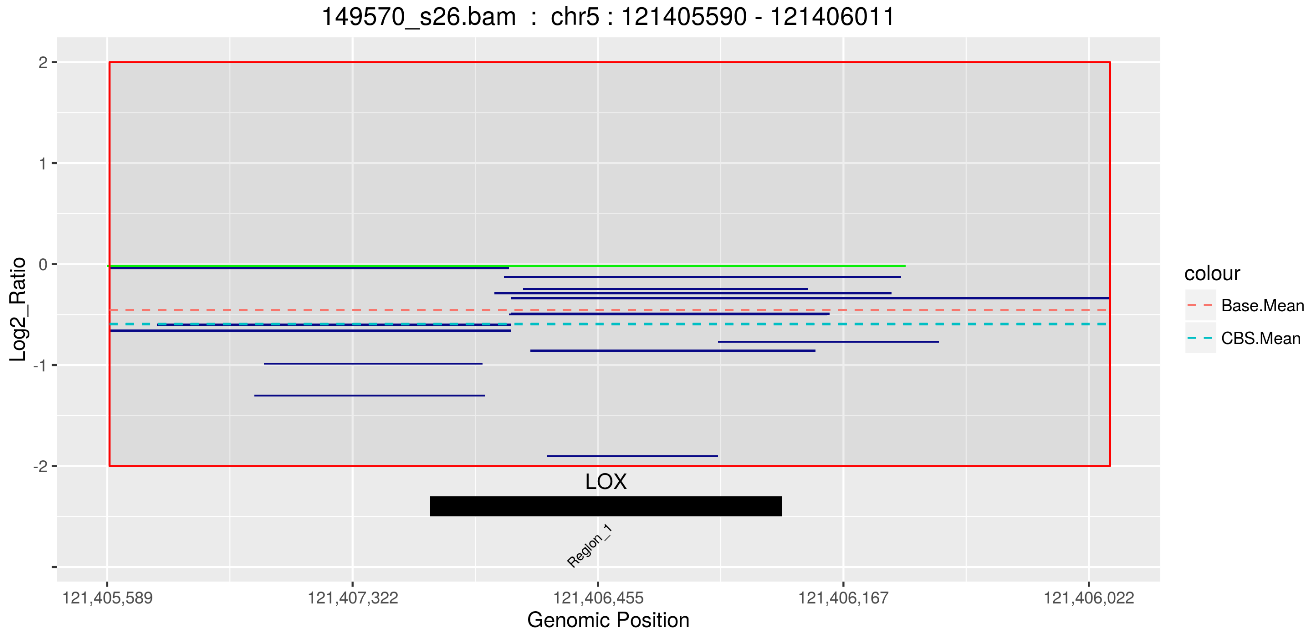

Figure S4.12: TAAD Panel-Batch3-T10: chr5-121405590-121406011-LOX-*Deletion*. The CNV region completely overlaps with the integral number of targeted exonic regions. Amplicons that are flanking (marked green) and within (marked blue) span the CNV region. None of flanking amplicons marked green and brown amplicons can be seen aligned to any part of the CNV regions. However, the orthogonal methods do not validate the deletion of this CNV region (*Region_1* of the *LOX* gene). The varAmpliCNV-DOF approach and k-mean clustering (k=5) correctly predicted it as TN.

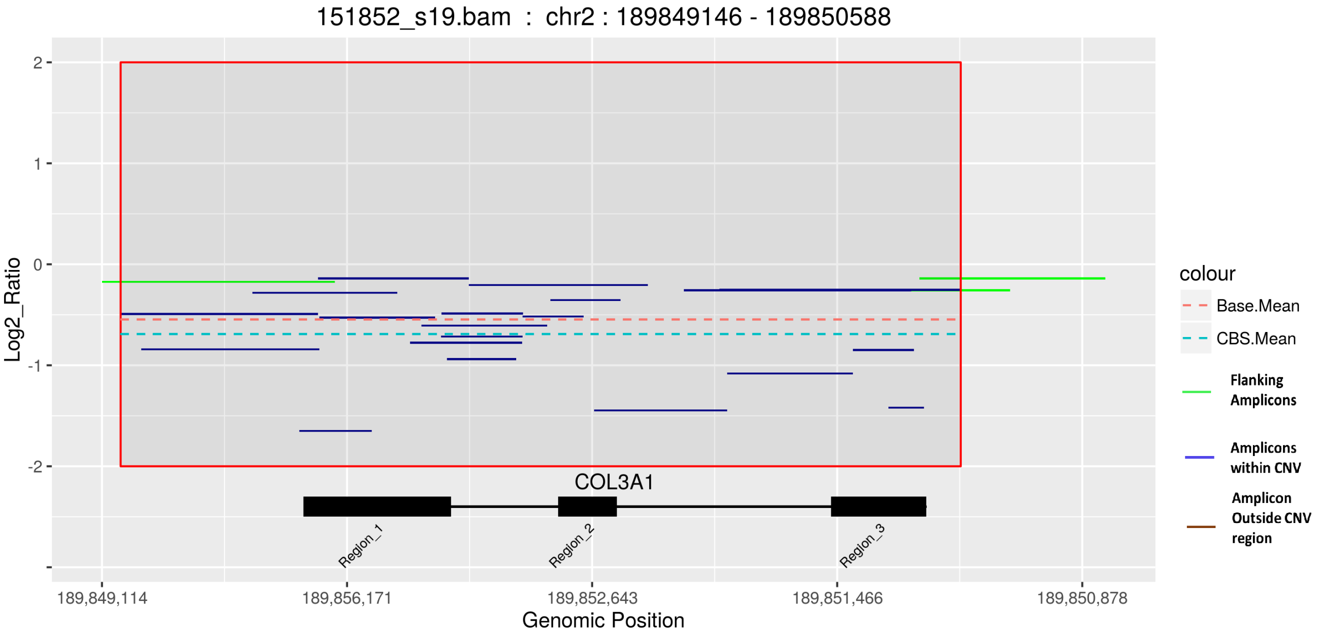

Figure S4.13: TAAD Panel-Batch4-T11: chr2-189849146-189850588-COL3A1-*Deletion*. The CNV region completely overlaps with the integral number of targeted exonic regions. Amplicons that are flanking (marked green) and within (marked blue) span the CNV region. None of flanking amplicons marked green and brown amplicons can be seen aligned to any part of the CNV regions. However, the orthogonal methods do not validate the deletion of this CNV region (*Region_1* to *Region_3*) of the *COL3A1* gene). It is classified as FP(DS approach and k-means clustering (BIC k=2)).

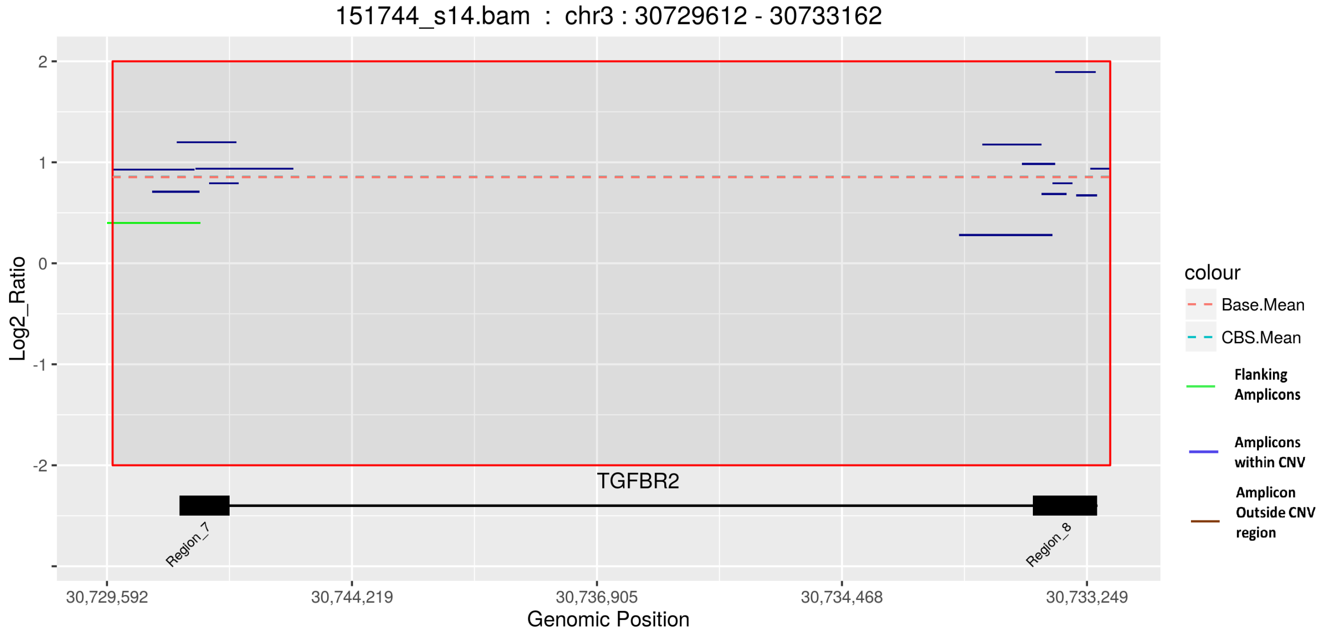

Figure S4.14: TAAD Panel-Batch4-T12: chr3-30729612-30733162-TGFBR2-*Duplication*. The CNV region completely overlaps with the integral number of targeted exonic regions. Amplicons that are flanking (marked green) and within (marked blue) span the CNV region. None of amplicons marked brown can be seen aligned to any other part of the CNV region. Additionally, orthogonal methods validate the duplication of this CNV region (as duplication from *Region_7* to *Region_8* of the *TGFBR2* gene). Hence, it is classified as TP.

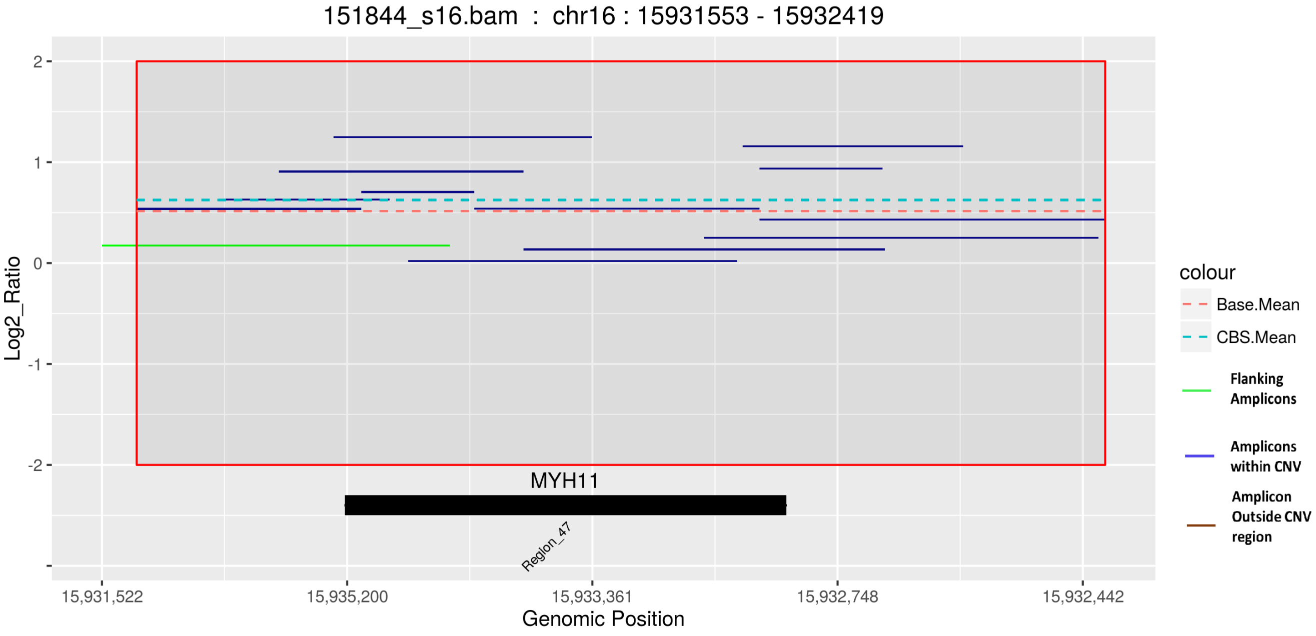

Figure S4.15: TAAD Panel-Batch4-T13: chr16-15931553-15932419-MYH11-*Duplication*. The CNV region completely overlaps with the integral number of targeted exonic regions. Amplicons that are *flanking* (marked green) and *within* (marked blue) span the CNV region. None of amplicons marked brown can be seen aligned to any other part of the CNV region. Additionally, orthogonal methods validate the duplication of this CNV region (as duplication of *Region_47* in the *MYH11* gene). It is classified as TP.

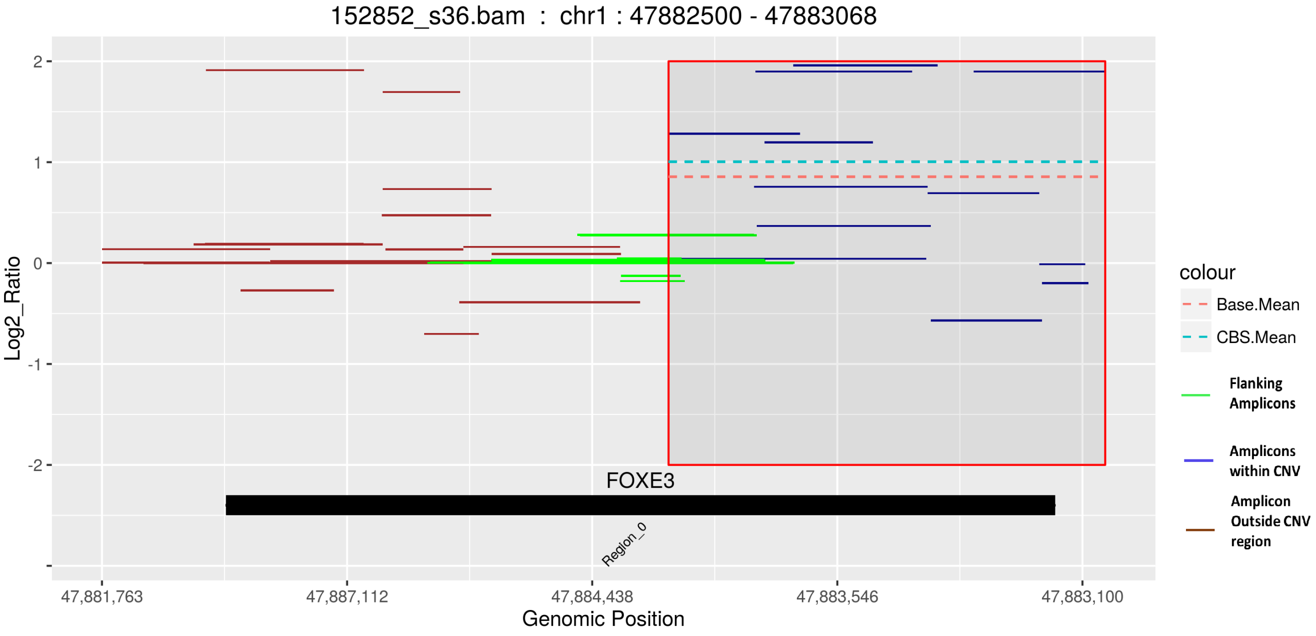

Figure S4.16: TAAD Panel-Batch4-T14: chr1-47882500-47883068-FOXE3-*Duplication*. Partial deletion of *Region_0* of the *FOXE3* gene. Amplicons *within* (marked blue) and *flanking* (marked green) span the CNV region. Small set of amplicons marked as brown align to other part of the targeted region of the gene. Additionally, orthogonal methods do not validate duplication of this region. It is classified as FP (DS approach and k-means clustering (BIC k=2)).

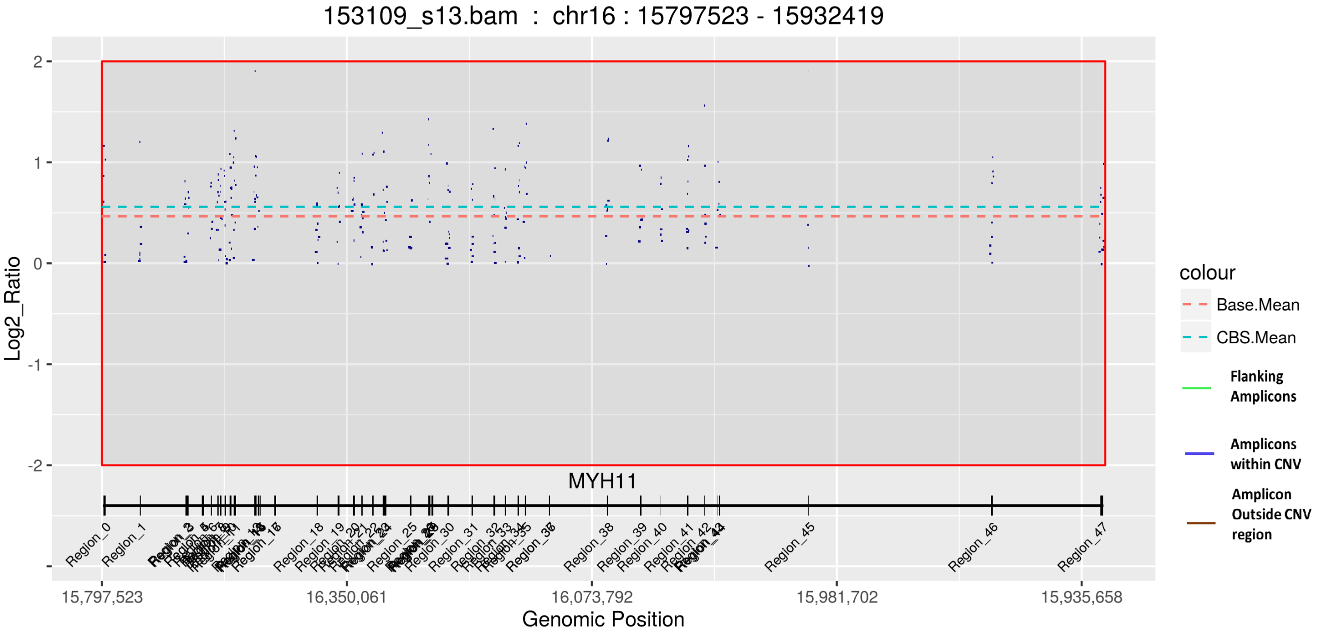

Figure S4.17: TAAD Panel-Batch4-T15: chr16-15797523-15932419-MYH11-*Duplication*. The CNV region completely overlaps with the integral number of targeted exonic regions. Amplicons that are *flanking* (marked green) and *within* (marked blue) span the CNV region. None of amplicons marked brown can be seen aligned to any other part of the targeted exonic region. Additionally, orthogonal methods validate the duplication of *Region_0* to *Region_47* in *MYH11* gene. It is classified as TP.

S4.1.3 Deafness panel

Deafness panel consisted of 145 genes analyzed blindfolded on 138 samples grouped in four batches. Application of DS-AOF approach with segmentation threshold (ST) interval (]-0.50,+0.50 [) same as derived from the TAAD panel resulted in 19 CNV segments as summarized by Figure 2D. Upon revealing ground truth, it was found that 3 TPs (D2, D11 and D14) were detected with current BT interval setting and one TP (D19) missed the interval. Rest, 15 CNV segments were candidates for TBC cnv lists subjected to validation by orthogonal methods.

Given the list is large, we formulate pruning/classification rules derived from previous section and visualization plots to trim down the list. Figure S4.14 to S4.31 present the visualization plots of 19 predicted CNV segments. We formulate following criteria for classifying predicted CNV segments as FPs, TBC and TPs:

1. Classify the predicted CNV segment as FP if it partially overlaps with integral number of targeted exonic region(s) (TER) of a given gene. A definitive pattern of amplicons marked blue and green can be found inside the CNV region and amplicons marked brown (outside CNV region) are seen aligned to remaining part of the TER.

2. Classify predicted CNV segment as candidate for TBC cnv lists if it completely overlaps with integral number of TER. All the amplicons (marked blue and green) are subjected to this region (i.e amplicons marked brown are not observed).

3. The predicted CNV region or candidate for TBC cnv lists that gets successfully validated by orthogonal methods are classified as TPs otherwise they are classified as FPs.

Visualization plot for data points D1- S4.14, D3- S4.16, D6- S4.19, D8- S4.21, D9- S4.22, D13- S4.26, D17- S4.30 and D18- S4.31 shows that they justify rule 1 as they correspond to partial deletion/duplication of single of integral number of TERs for a given gene. They can be easily classified as FPs and discarded from candidate list of TBC cnv lists.

Similarly, visualizations corresponding to 3 TP datapoints are: D2- S4.15, D11- S4.24 and D14- S4.27. From these visualization it can be summarized that rule 2 and 3 are justified. Finally, seven datapoint D4- S4.17, D5- S4.18, D7- S4.20, D10- S4.23, D12- S4.25, D15- S4.28 and D16- S4.29 justify rule 2 and are hence candidate for TBC cnv lists subjected to experimental validation. Overall, DS-AOF approach resulted in 15/19 CNV segments but by above pruning/classification rule and visualization helped in trimming the list to 7 segments as candidate for TBC cnv lists. Also, the lone false negative (FN) data point D19- S4.32 also justifies rule 2 and is experimentally validated by orthogonal method. But, this data point was filtered by DS-AOF approach as it did not pass through ST interval]-0.50,0.50[. Hence it was not selected as candidate segments for TBC cnv list and no visualization based filtering was applied to it.

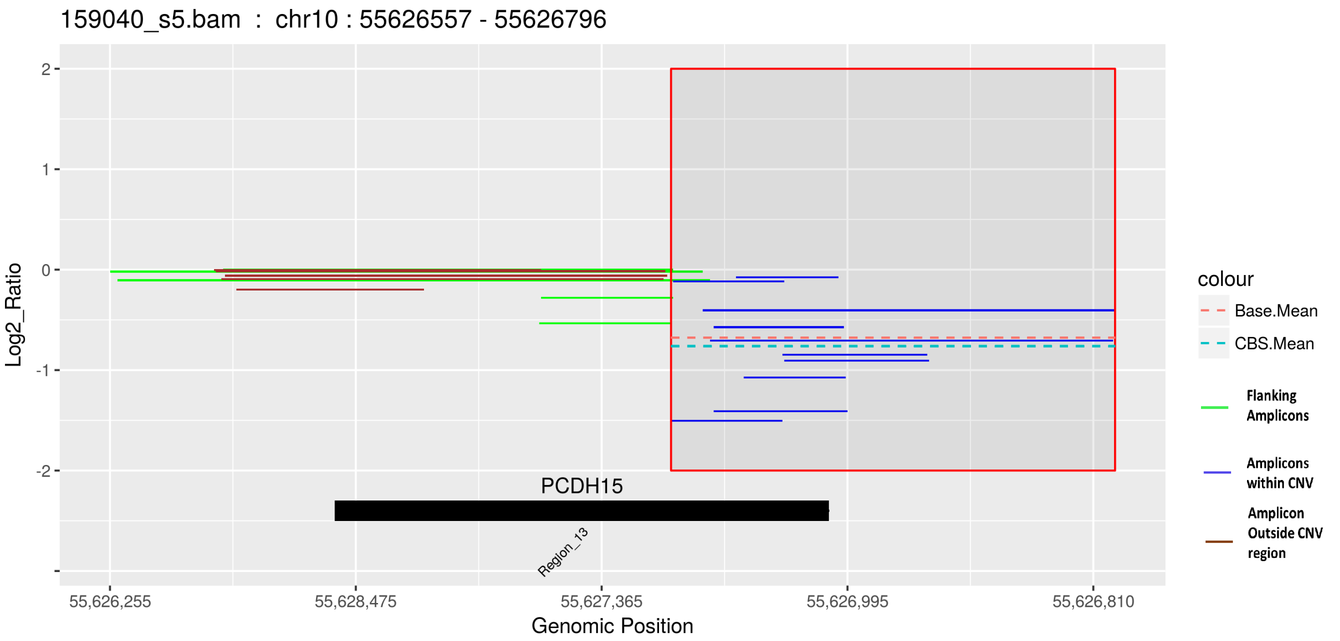

Figure S4.14: Deafness Panel-Batch1-D1: chr10-55626557-55626796-PCDH15-*Deletion*. Partial deletion of *Region_13* of PCDH15 gene. Amplicons *within* (marked blue) and *flanking* (marked green) span the CNV region (red colour box). Small set of amplicons marked as brown align to other part of *Region_13* of *PCDH15* gene. It is classified as FP.

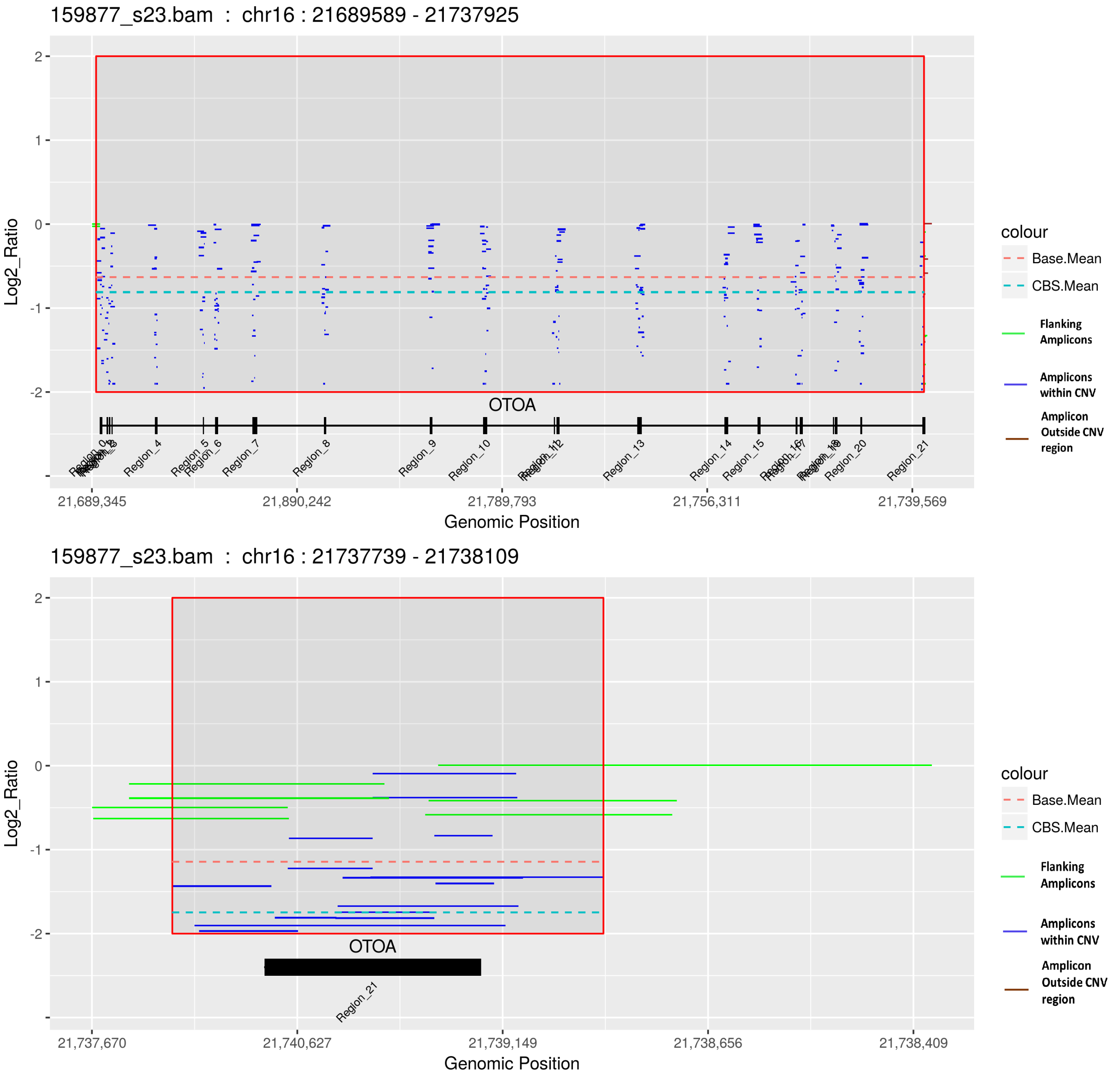

Figure S4.15: Deafness Panel-Batch1-D2: chr16-21737739-21738109-OTOA -*Deletion*. The CNV region completely overlaps with the integral number of targeted exonic regions. Amplicons that are marked blue and green span the CNV region. Additionally, orthogonal methods validate true deletion of *OTOA*gene. It is classified as TP.

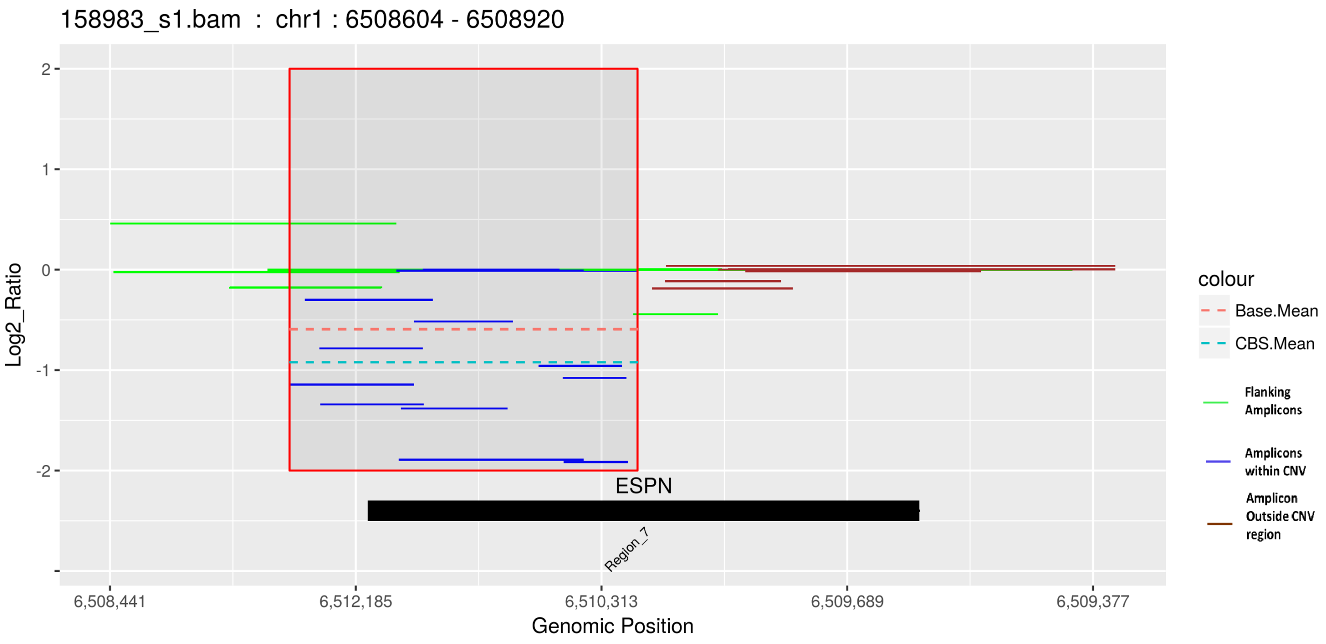

Figure S4.16: Deafness Panel-Batch1-D3: chr1-6508604-6508920-ESPN-*Deletion*. Partial deletion of *Region_7* of the gene. Amplicons *within* (marked blue) and *flanking* (marked green) span the CNV region. Small set of amplicons marked as brown align to other part of *Region_7* of *ESPN* gene. Can be classified as FP.

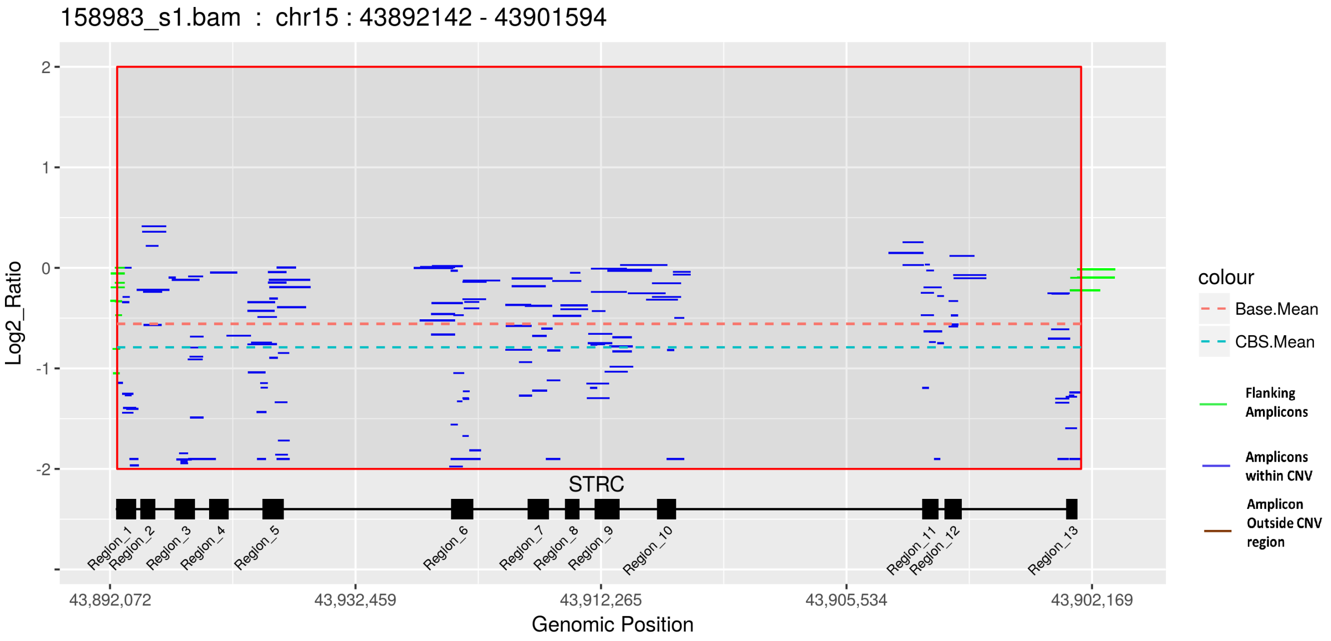

Figure S4.17: Deafness Panel-Batch1-D4: chr1-43892142-43901594-STRC-*Deletion*. The CNV region completely overlaps with the integral number of targeted exonic regions. Amplicons that are marked blue and green span the CNV region. Candidate for further test (TBC) by orthogonal methods.

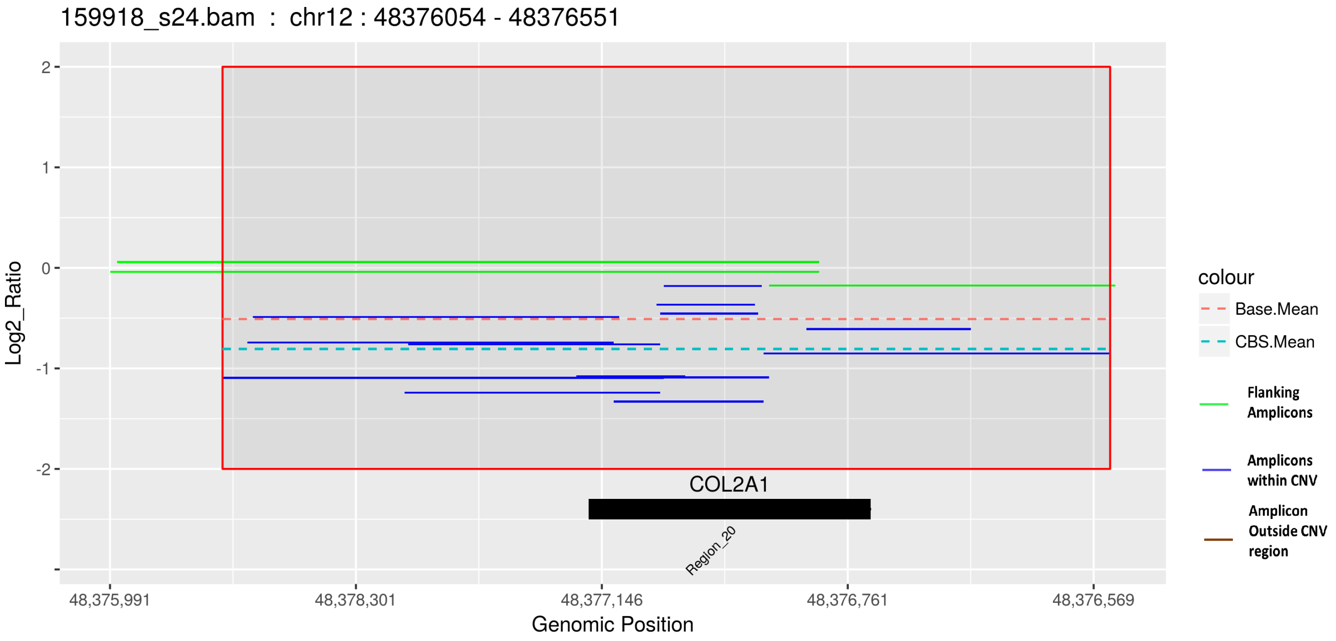

Figure S4.18: Deafness Panel-Batch1-D5: chr12-48376054-48376551-COL2A1-*Deletion*. The CNV region completely overlaps with the integral number of targeted exonic regions. Amplicons that are marked blue and green span the CNV region. Candidate for further test (TBC) by orthogonal methods.

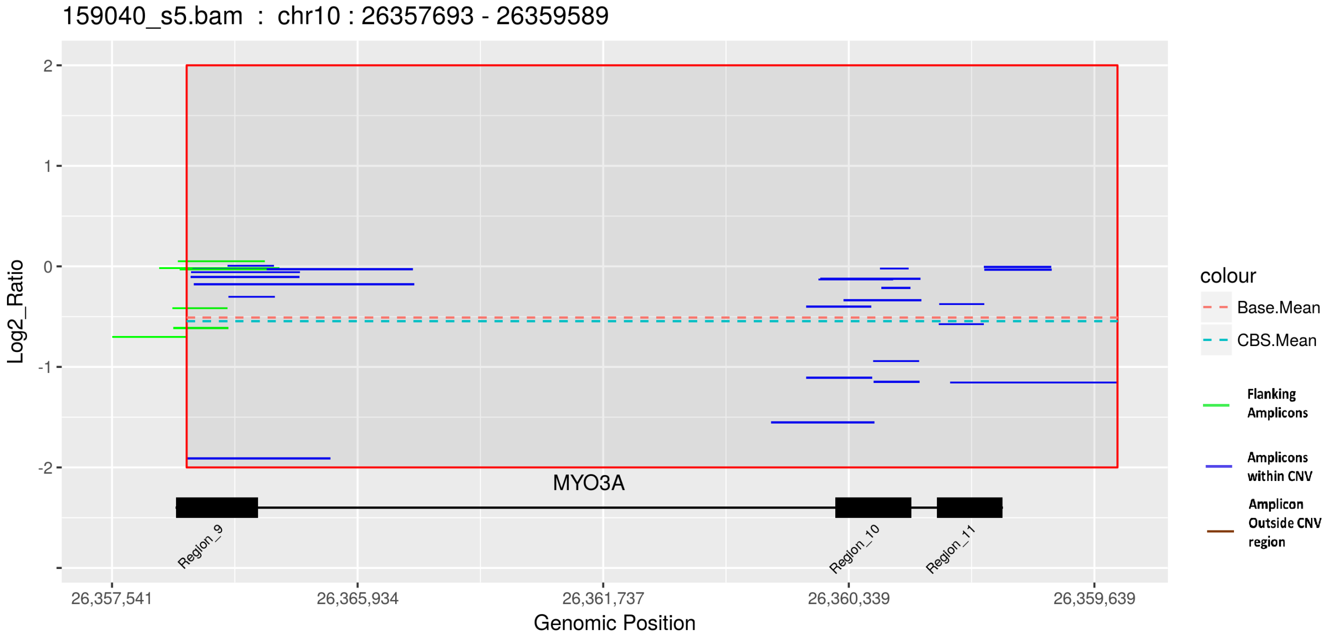

Figure S4.19: Deafness Panel-Batch1-D6: chr10-2637693-26359589-MYO3A-*Deletion*. The CNV region partially overlaps with *Region_9* of *MYO3A* gene. It is classified as FP.

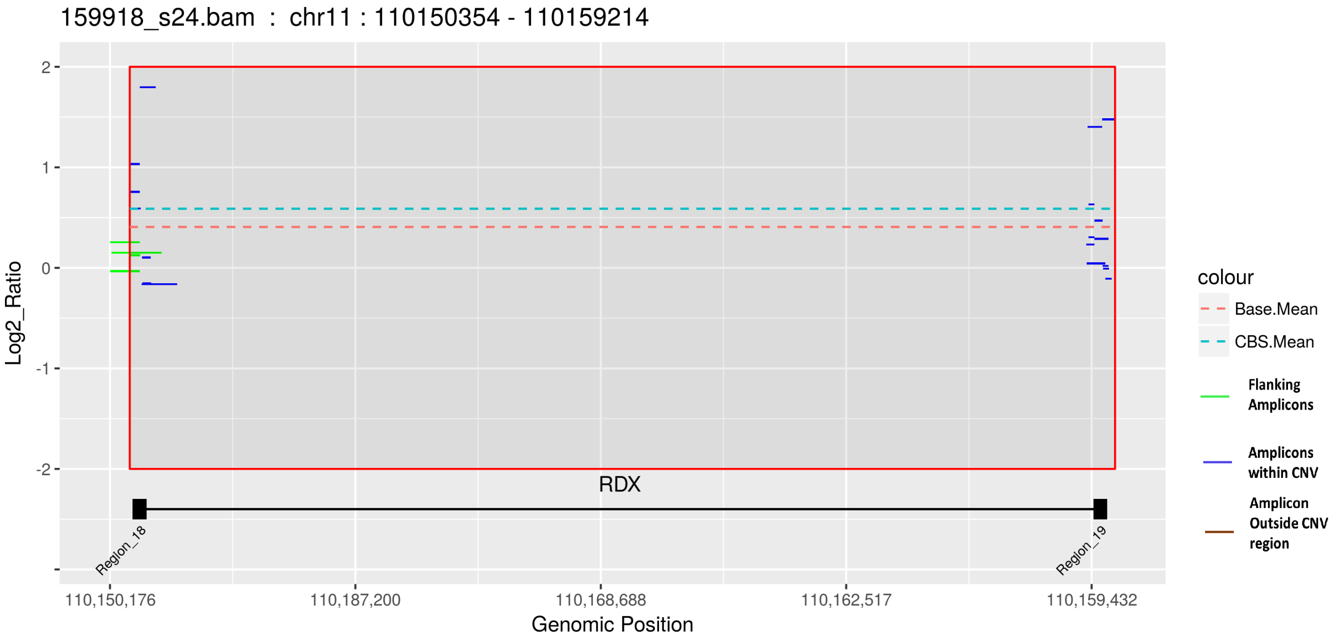

Figure S4.20: Deafness Panel-Batch1-D7: chr11-110150354-110159214-RDX-*Duplication*. The CNV region completely overlaps with the integral number of targeted exonic regions. Amplicons that are marked blue and green span the CNV region. Candidate for further test (TBC) by orthogonal methods.

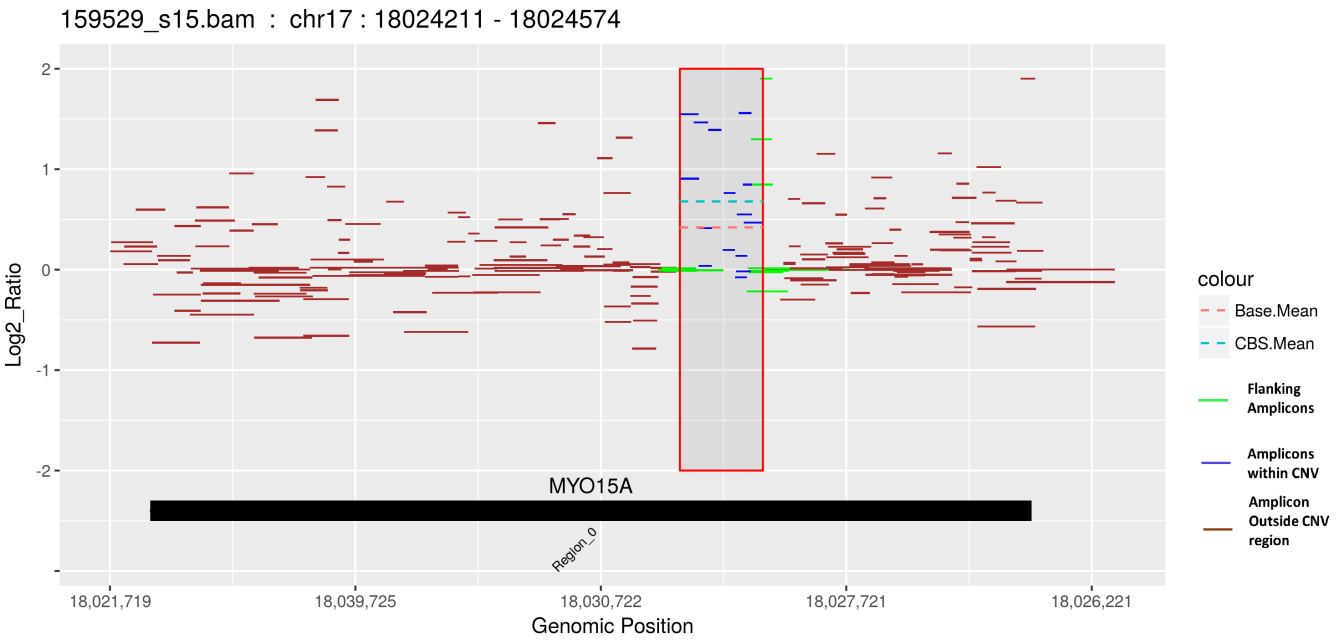

Figure S4.21: Deafness Panel-Batch1-D8: chr17-18024211-18024574-MYO15A-*Duplication*. Partial duplication of *Region_0* of the gene. Amplicons *within* (marked blue) and *flanking* (marked green) span the CNV region. Large set of amplicons marked as brown align to other part of *Region_0* of *MYO15A* gene. Can be classified as FP.

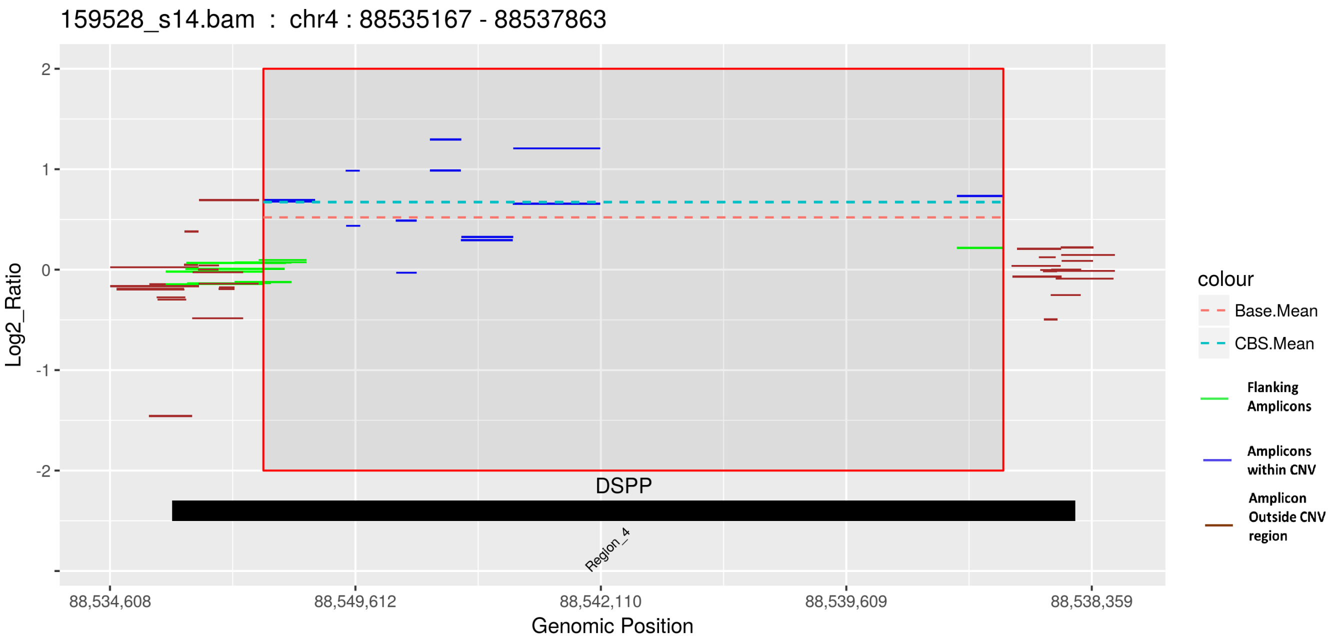

Figure S4.22: Deafness Panel-Batch1-D9: chr4-88535167-88537863-DSPP-*Duplication*. Partial duplication of *Region_4* of the gene. Amplicons *within* (marked blue) and *flanking* (marked green) span the CNV region. Small set of amplicons marked as brown align to other part of *Region_4* of *DSPP* gene. Can be classified as FP.

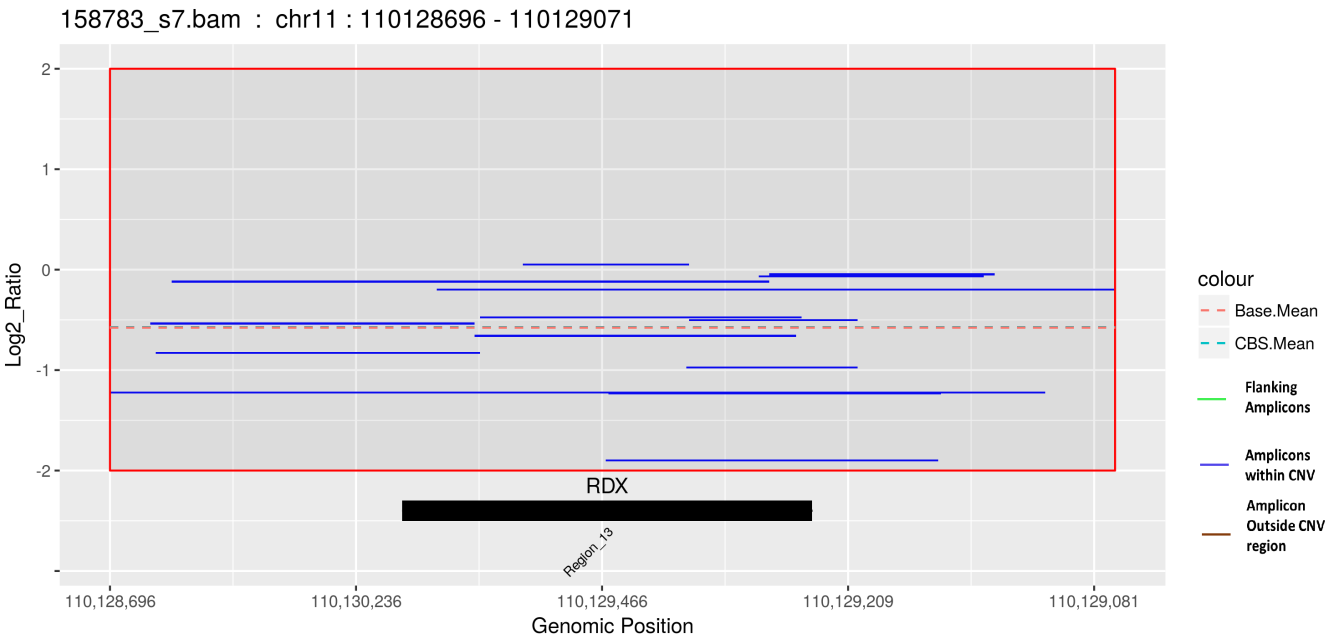

Figure S4.23: Deafness Panel-Batch2-D11: chrX-43817808-43818082-NDP-AS1-*Deletion* (top);chrX-82763008-82764806-POU3F4-*Deletion*(bottom). (Top) The CNV region completely overlaps with the integral number of targeted exonic regions. Amplicons that are marked blue and green span the CNV region. Candidate for further test (TBC) by orthogonal methods.

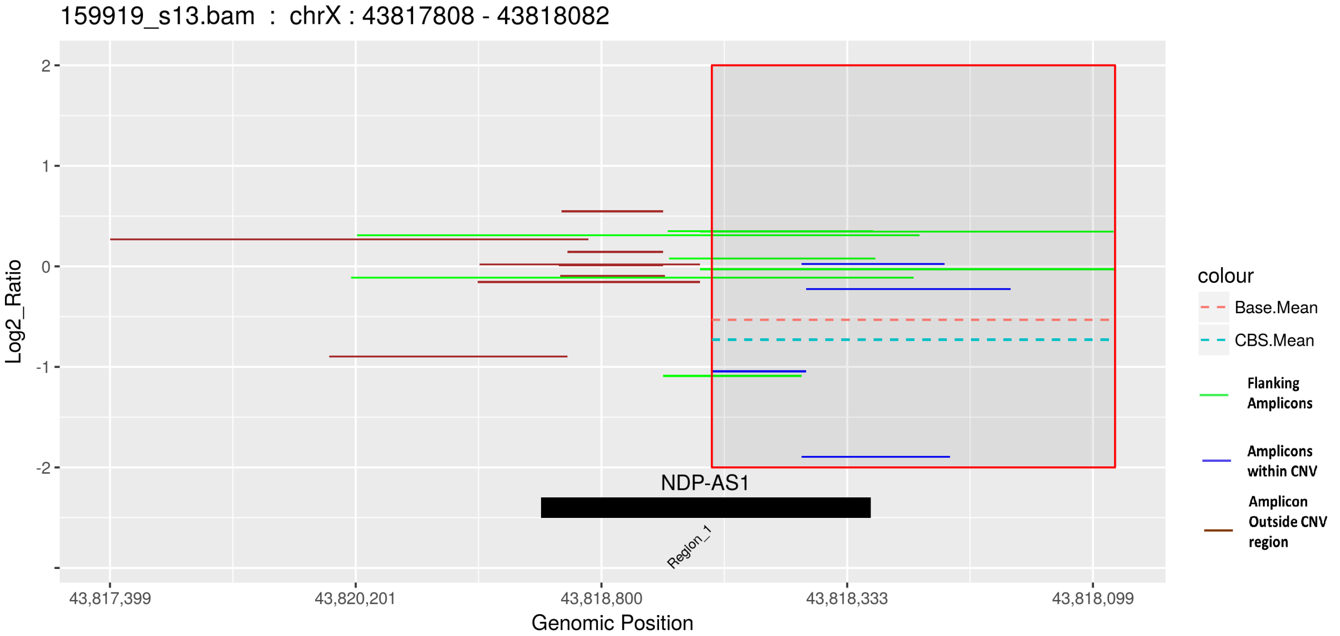

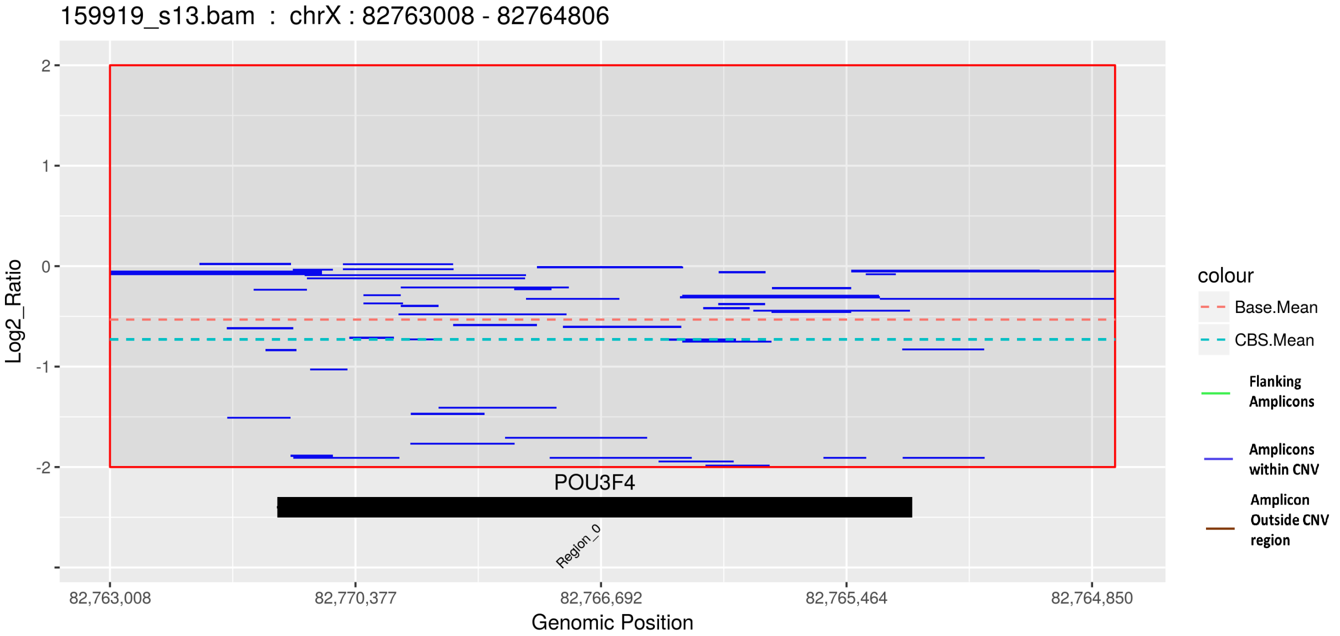

Figure S4.24: Deafness Panel-Batch2-D11: chrX-43817808-43818082-NDP-AS1-*Deletion* (top);chrX-82763008-82764806-POU3F4-*Deletion*(bottom). (Top) Partial duplication of *Region_1* of the *NDP-AS1* gene. Amplicons *within* (marked blue) and *flanking* (marked green) span the CNV region. Small set of amplicons marked as brown align to other part of *Region_1* of *NDP-AS1* gene. Can be classified as FP. (Bottom) The CNV region completely overlaps with the integral number of targeted exonic regions of *POU3F4* gene. Amplicons that are marked blue and green span the CNV region. Additionally, it was validated by orthogonal method. It can be classified as TP.

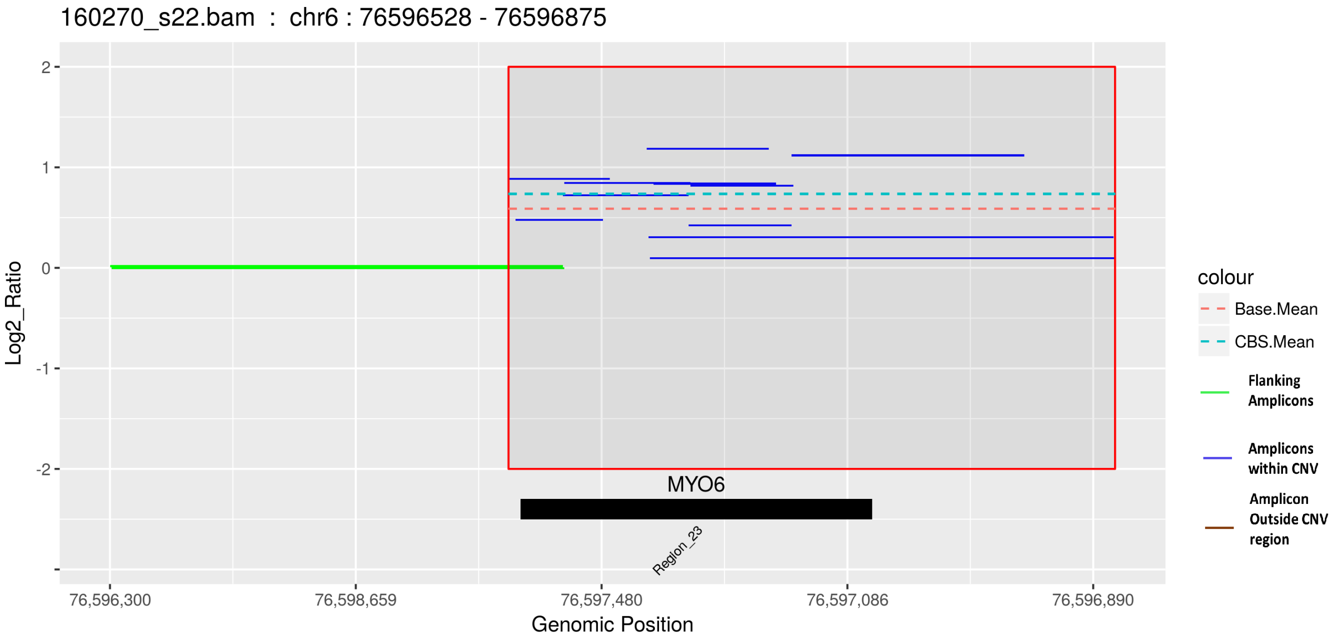

Figure S4.25: Deafness Panel-Batch2-D12: chr6-76596528-76596875-MYO6-*Duplication.*The CNV region completely overlaps with the integral number of targeted exonic regions. Amplicons that are marked blue and green span the CNV region. Candidate for further test (TBC) by orthogonal methods.

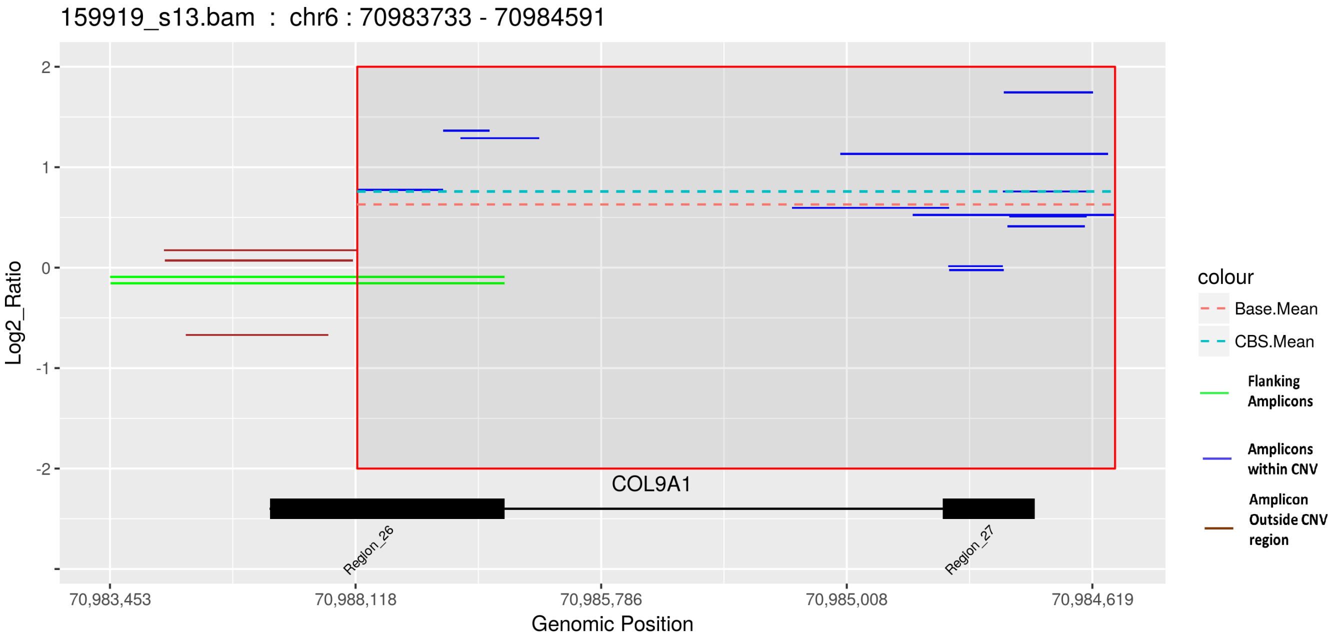

Figure S4.26: Deafness Panel-Batch2-D13: chr6-70983733-70984591-COL9A1-*Duplication*. Partial duplication of *Region_26* of *COL9A1* gene. Amplicons *within* (marked blue) and *flanking* (marked green) span the CNV region. Small set of amplicons marked as brown align to other part of *Region_26*. It is classified as FP.

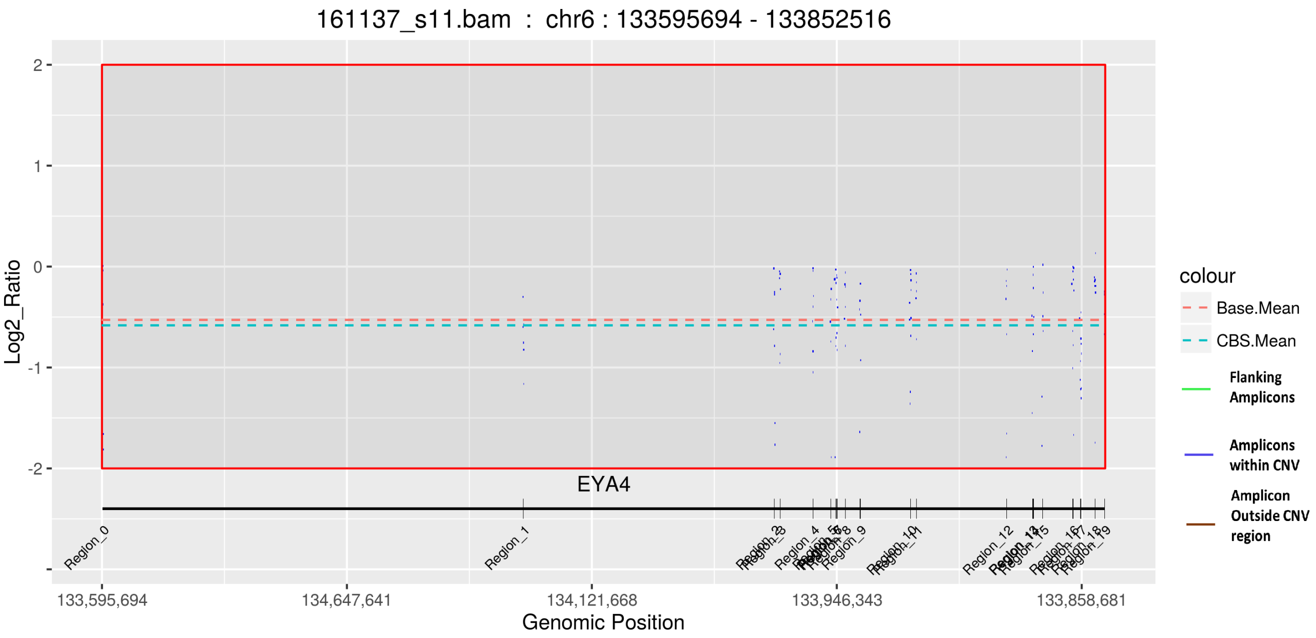

Figure S4.27: Deafness Panel-Batch3-D14: chr6-133595694-133852516-EYA4-*Deletion*. The CNV region completely overlaps with the integral number of targeted exonic regions of *EYA4* gene. Amplicons that are marked blue and green span the CNV region. Additionally, it was validated by orthogonal method. It is classified as TP.

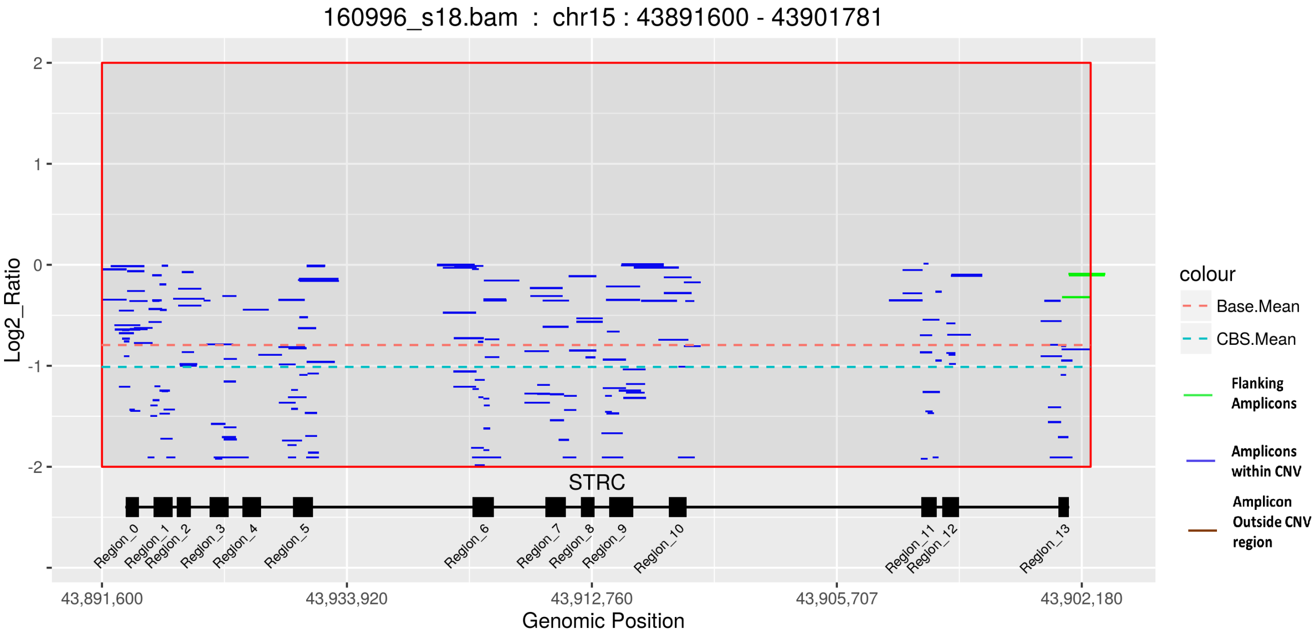

Figure S4.28: Deafness Panel-Batch4-D15: chr15-43891600-43901781-STRC-*Deletion*. The CNV region completely overlaps with the integral number of targeted exonic regions. Amplicons that are marked blue and green span the CNV region. Candidate for further test (TBC) by orthogonal methods.

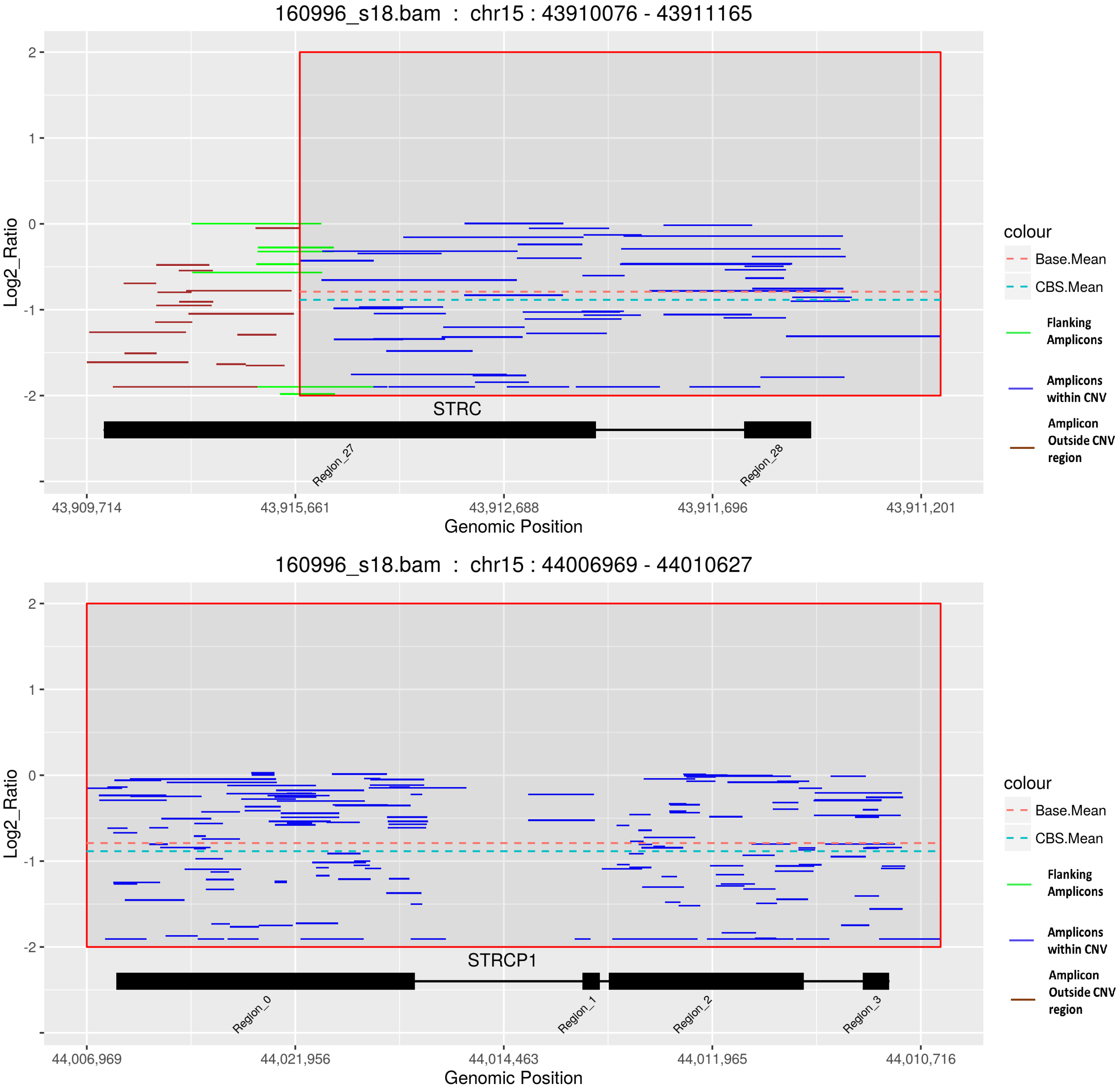

Figure S4.29: Deafness Panel-Batch4-D16: chr15-43910076-43911165-STRC-*Deletion* (top); chr15-44006969-44010627-STRCP1-*Deletion* (bottom). (Top) Partial deletion of *Region_27* of the *STRC* gene. Amplicons within (marked blue) and flanking (marked green) span the CNV region. Small set of amplicons marked as brown align to other part of *Region_27*. It is classified as FP. (Bottom) The CNV region completely overlaps with the integral number of targeted exonic regions. Amplicons that are marked blue and green span the CNV region. Candidate for further test (TBC) by orthogonal methods.

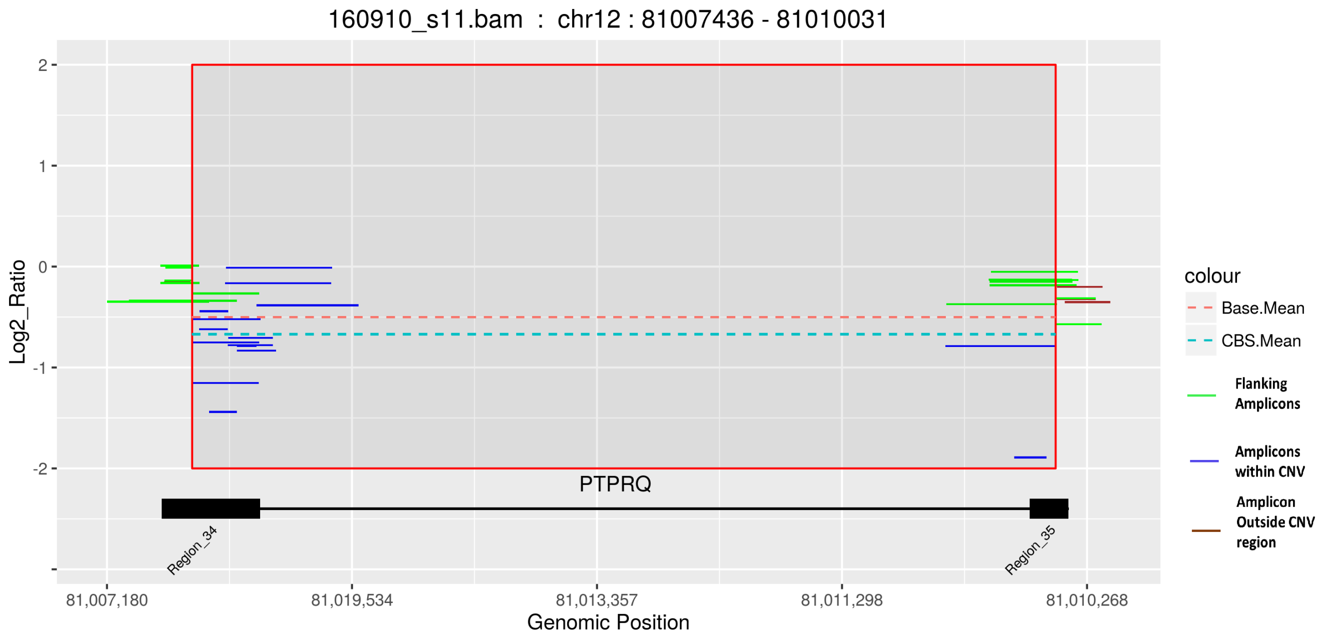

Figure S4.30: Deafness Panel-Batch4-D17: chr12-81007436-81010031-PTPRQ-*Deletion*. Partial deletion of *Region_34* and *Region_35* of *PTPRQ* gene. Amplicons *within* (marked blue) and *flanking* (marked green) span the CNV region. Small set of amplicons marked as brown align to other part of *Region_35*. It is classified as FP.

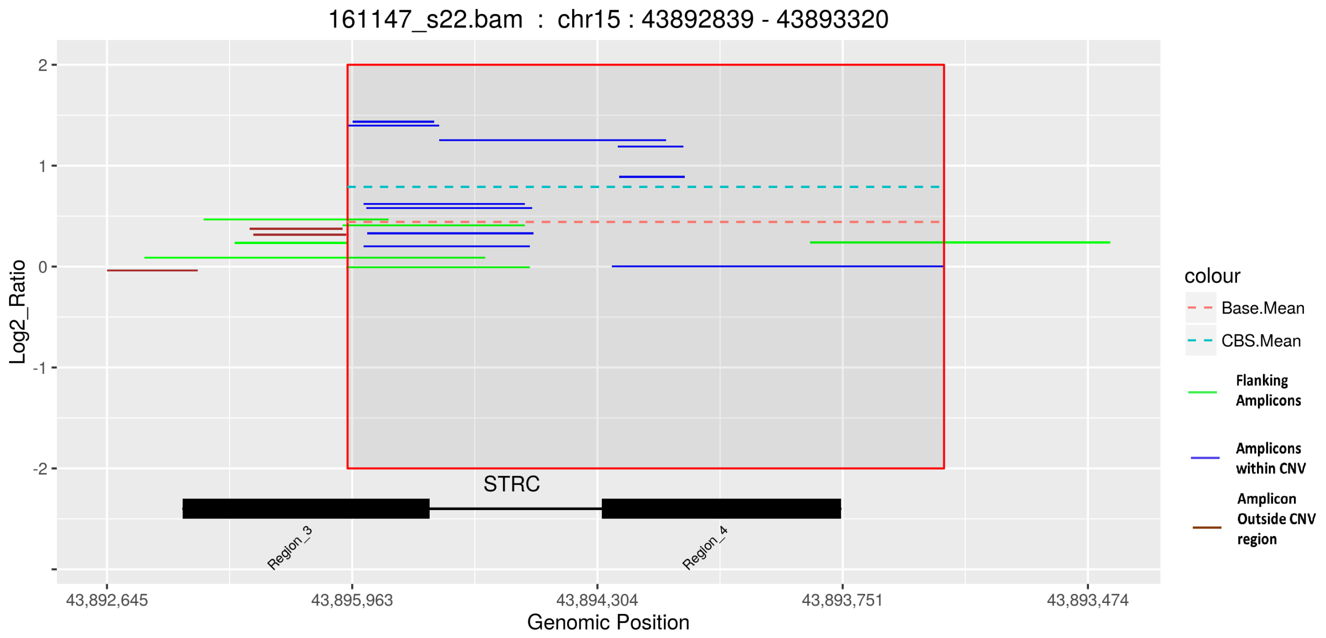

Figure S4.31: Deafness Panel-Batch4-D18: chr15-43892839-43893320-STRC-*Duplication*. Partial duplication of *Region_3* and *Region_4* of *STRC* gene. Amplicons *within* (marked blue) and *flanking* (marked green) span the CNV region. Small set of amplicons marked as brown align to other part of *Region_3*. It is classified as FP.

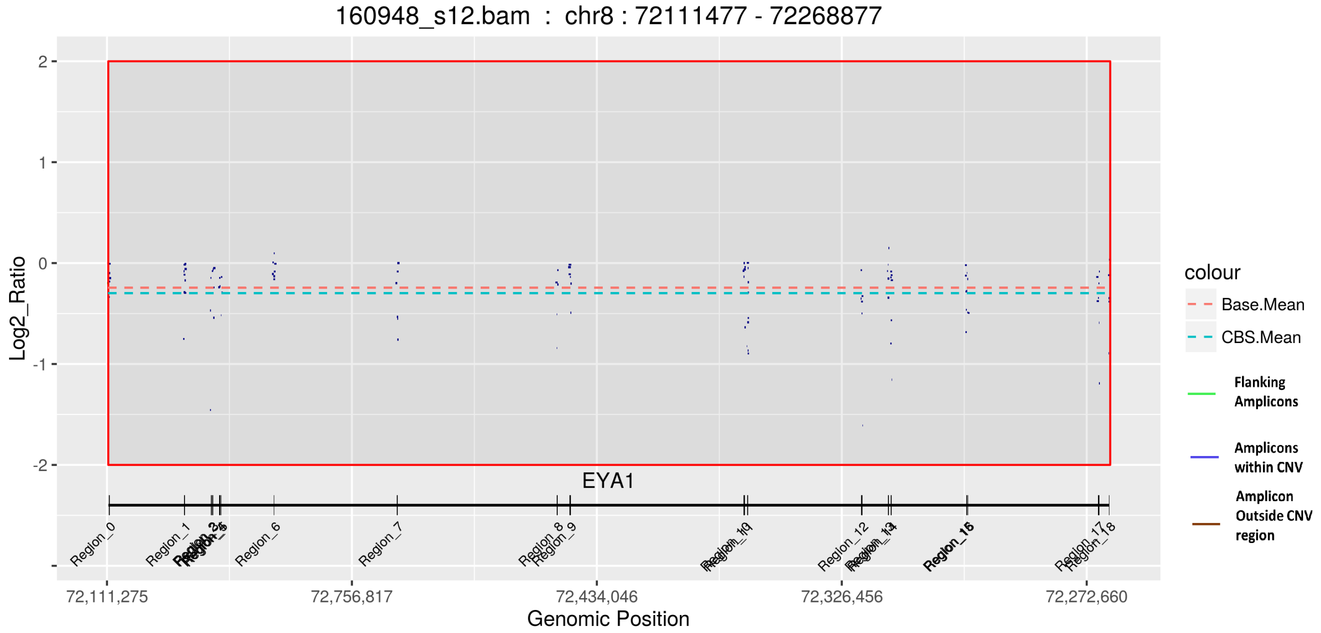

Figure S4.32: Deafness Panel-Batch4-D19: chr8-72111477-72268877-EYA1-*Deletion*. The CNV region completely overlaps with the integral number of targeted exonic regions (TER) of *EYA1* gene. Amplicons that are marked blue span the CNV region. Experimental validation by orthogonal methods as TP. But, could not get detected by derived ST interval]-0.50, 50[and k-means clustering (BIC k=2). It is classified as false negative (FN).

S5 One dimensional k-means clustering of CNV segments

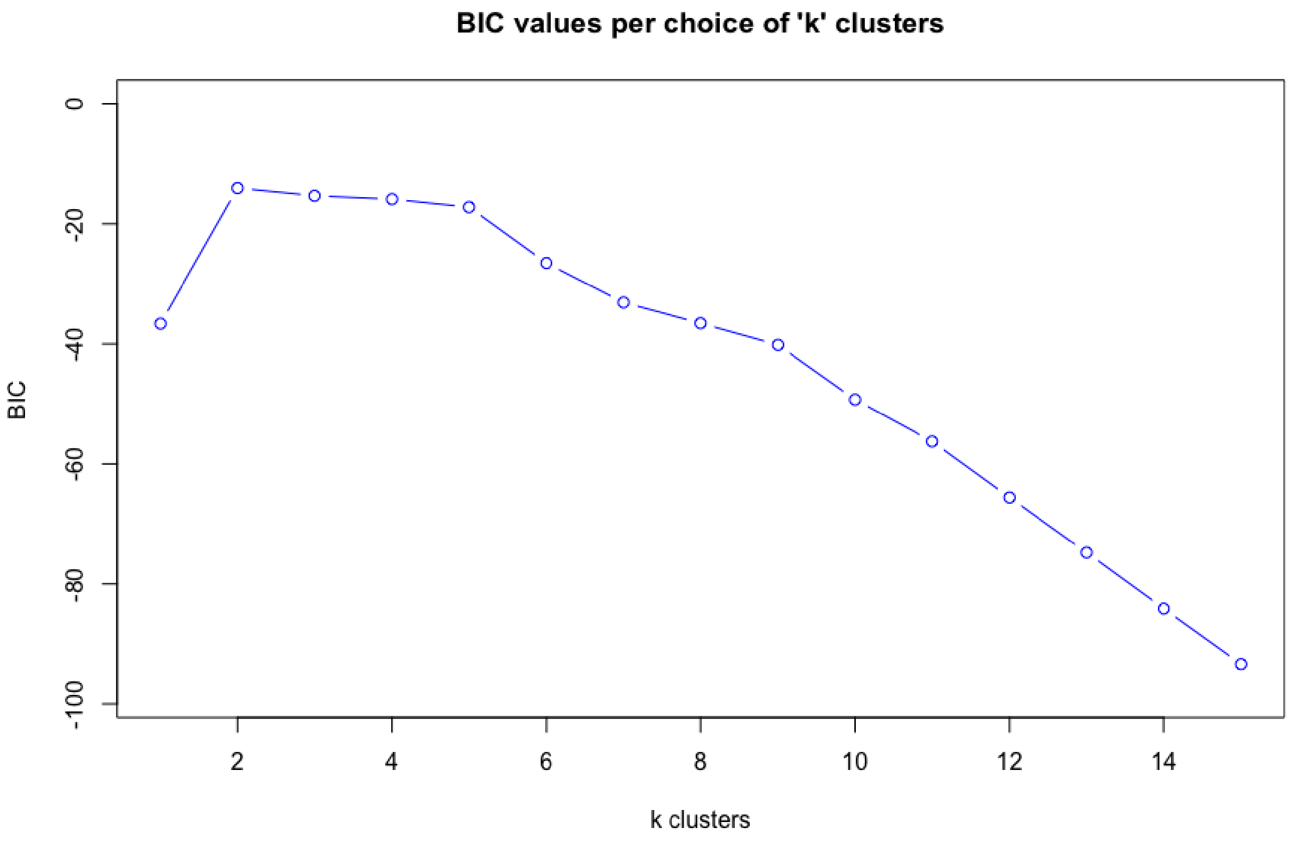

Figure S5.1: BIC value vs number of *k* clusters : Plot of Bayesian information criteria (BIC) score against choice of k clusters of 15 CNV segments of TAAD panel obtained from DS approach using (1-dimensional kmeans clustering). The CNV segments were cluster using the $log_2 R$ score. The maximum BIC is obtained for k=2

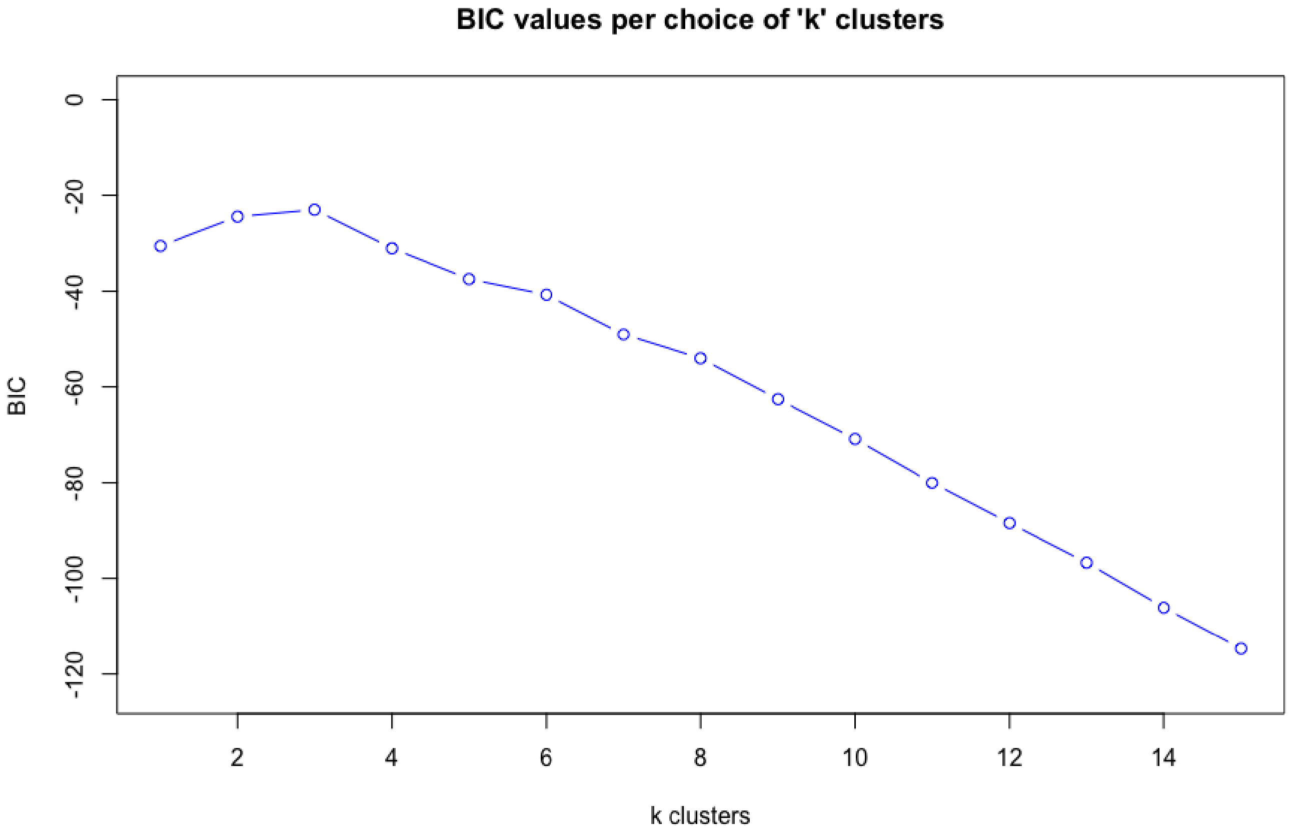

Figure S5.2: BIC value vs number of *k* clusters : Plot of Bayesian information criteria (BIC) score against choice of k clusters of 15 CNV segments of TAAD panel obtained from DS-AOF approach using (1-dimensional kmeans clustering). The CNV segments were cluster using the average log_2R score obtained using AOF approach. The maximum BIC is obtained for k=3

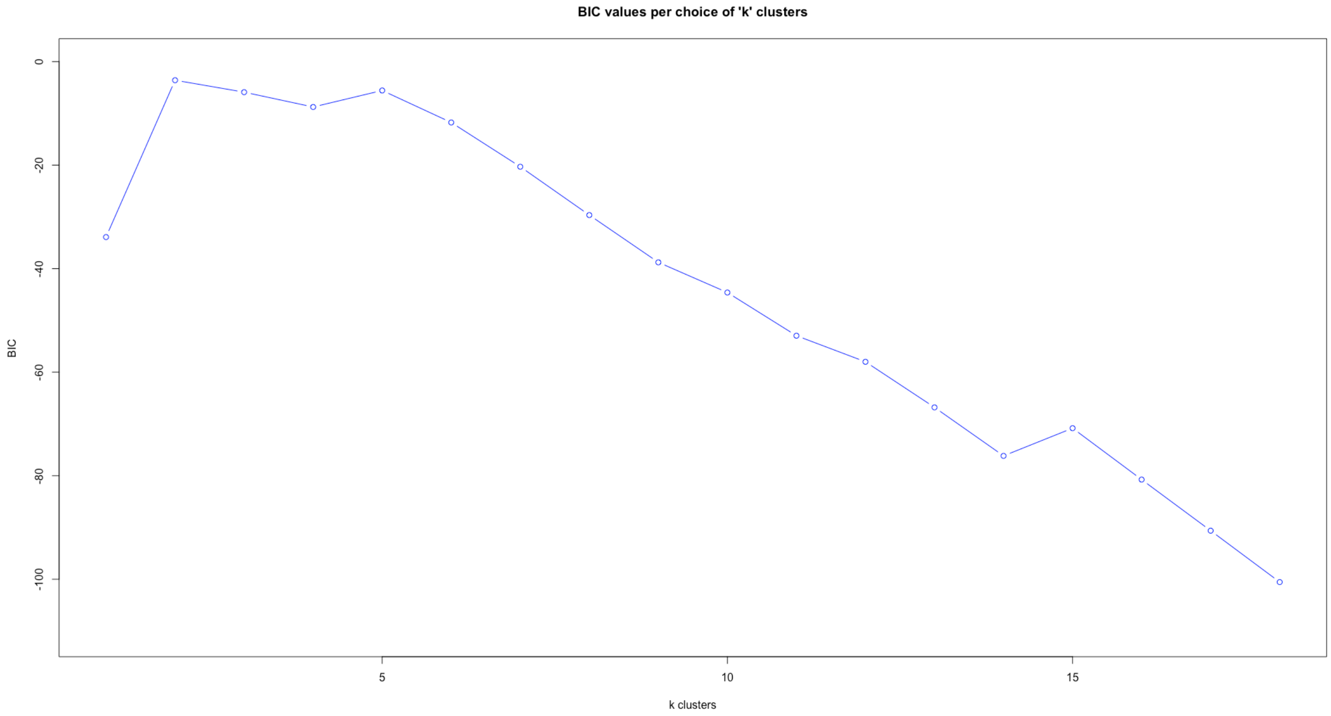

Figure S5.3: BIC value vs number of *k* clusters : Plot of Bayesian information criteria (BIC) score against choice of k clusters of 15 CNV segments of Deafness panel obtained from DS-AOF approach using (1-dimensional kmeans clustering). The CNV segments were cluster using the average log_2R score obtained using AOF approach The maximum BIC is obtained for k=2
